# A contamination focused approach for optimizing the single-cell RNA-seq experiment

**DOI:** 10.1016/j.isci.2023.107242

**Published:** 2023-06-29

**Authors:** Deronisha Arceneaux, Zhengyi Chen, Alan J. Simmons, Cody N. Heiser, Austin N. Southard-Smith, Michael J. Brenan, Yilin Yang, Bob Chen, Yanwen Xu, Eunyoung Choi, Joshua D. Campbell, Qi Liu, Ken S. Lau

**Affiliations:** 1 Epithelial Biology Center, Vanderbilt University Medical Center, Nashville, TN, USA; 2 Department of Cell and Developmental Biology, Vanderbilt University School of Medicine, Nashville, TN, USA; 3 Program in Chemical and Physical Biology, Vanderbilt University School of Medicine, Nashville, TN, USA; 4 McDonnell Genome Institute and Department of Medicine, Washington University in St. Louis, St. Louis, MO, USA; 5 1CellBio, Inc., Watertown, MA, USA; 6 Department of Surgery, Vanderbilt University Medical Center, Nashville, TN, USA; 7 Section of Computational Biomedicine, Department of Medicine, Boston University School of Medicine, Boston, MA, USA; 8 Department of Biostatistics and Center for Quantitative Sciences, Vanderbilt University Medical Center, Nashville, TN, USA

**Keywords:** Computational bioinformatics, Transcriptomics, Biology experimental methods

## Abstract

Droplet-based single-cell RNA-seq (scRNA-seq) data are plagued by ambient contaminations caused by nucleic acid material released by dead and dying cells. This material is mixed into the buffer and is co-encapsulated with cells, leading to a lower signal-to-noise ratio. Although there exist computational methods to remove ambient contaminations post-hoc, the reliability of algorithms in generating high-quality data from low-quality sources remains uncertain. Here, we assess data quality before data filtering by a set of quantitative, contamination-based metrics that assess data quality more effectively than standard metrics. Through a series of controlled experiments, we report improvements that can minimize ambient contamination outside of tissue dissociation, via cell fixation, improved cell loading, microfluidic dilution, and nuclei versus cell preparation; many of these parameters are inaccessible on commercial platforms. We provide end-users with insights on factors that can guide their decision-making regarding optimizations that minimize ambient contamination, and metrics to assess data quality.

## Highlights

• Quantitative metrics to assess ambient contaminations in scRNA-seq data
• Metrics are applied to prefiltered datasets to evaluate data quality at large scale
• Nuclei preparation has minimal effect on reducing contamination
• Cell loading mechanism has the biggest effect on ambient contamination

## Graphical abstract

**Figure:**
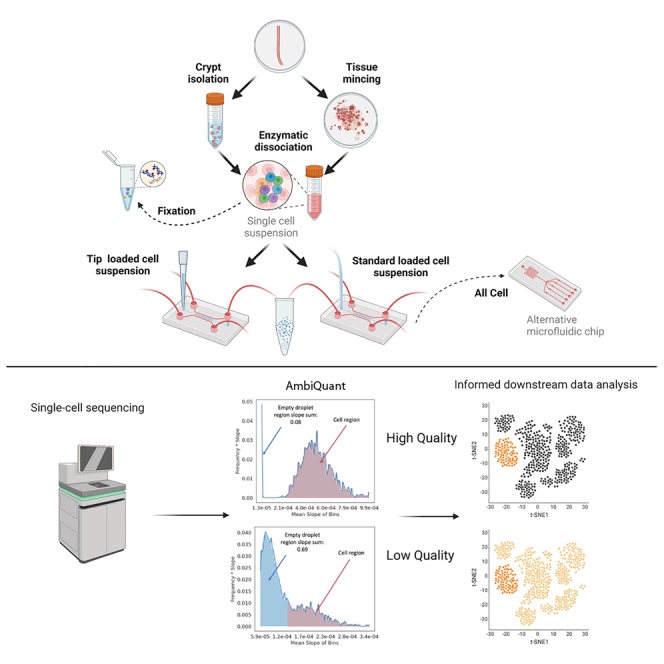

## Introduction

Single-cell RNA sequencing (scRNA-seq) is a technique that allows for the investigation of genome-scale gene expression in thousands of individual cells, facilitating the deconvolution of tissue heterogeneity and population dynamics. The are many scRNA-seq platforms that employ various strategies to partition single cells. Plate-based methods involve the deposition and lysis of individual cells in multiwell plates.^1^^,^^2^^,^^3^ Microfluidic-based methods employ microfluidic chambers and arrays to isolate and process single cells.^4^ Templated emulsions and combinatorial indexing are innovative methods that do not require specialized equipment to barcode single cells^5^^,^^6^^,^^7^ Lastly, spatial sequencing enables gene expression while maintaining spatial resolution.^8^ The most popular scRNA-seq platforms by far are droplet-based methods, which involve microfluidic encapsulation of cells and barcoded capture oligonucleotides in oil emulsions, that ultimately enable sequencing reads to be assigned to each droplet or cell.^9^^,^^10^^,^^11^ For droplet-based methods, because of the low cellular loading required to avoid two or more cells captured in an individual droplet, most droplets are devoid of cells and ideally only contain loading buffer and RNA-capture beads. However, this is often not the case because stress encountered by cells during single-cell processing induces cell death and leakage, leading to deposition of ambient RNA into the loading buffer. This RNA is either co-captured with cells into droplets or into empty droplets themselves.^12^ Ambient RNA contamination lowers effective sequencing read depth and, more importantly, contributes to an insidious signal that masks biological signals and confounds downstream biological interpretation.

The dissociation of tissues into single cell suspensions is a well-known cause of ambient contamination. Thus, a variety of tissue dissociation strategies have been developed, many of which were optimized for cell viability specific to tissue and cell types. For instance, van der Wijst et al. developed a one-step collagenase dissociation protocol for gut mucosal biopsies.^13^ A listing of many of these protocols matched to tissue types was presented by Regev and colleagues.^14^ Many of these protocols generate highly viable cells coming out of dissociation, as assessed by flow cytometry and live/dead dye visualization. However, they do not address the continuous stresses that cells are exposed to downstream of dissociation before and during the encapsulation process. Single cells in suspensions removed from their native tissue niches are often more prone to cell death.^15^ For processing death-prone tissues, single-nucleus RNA-seq (snRNA-seq) has been developed.^16^ Because nuclei are not cells, the prevalent thought in the field is that they are resistant to typical stresses that induce cell death. However, substantial amounts of cytoplasmic RNA and ribosomes adhere to the surfaces of isolated nuclei.^14^ In addition, the lysis buffers used for cell lysis may also damage nuclei and result in leakage RNAs. An area that has received relatively little attention in droplet-based approaches is the investigation into the fluidic technologies themselves, which are often viewed as a 'black box' by most end-users.

Another missing element is a quantitative method to evaluate data quality in the context of contamination. Although the ambient contamination issue is well-known in the field, data quality is still often evaluated using standard quality control (QC) metrics, mainly focused on the number of cells, genes, total transcripts, and mitochondrial transcripts recovered (Hong et al., 2022^17^), which cannot identify ambient contamination. Tools exist to leverage ambient contamination for filtering cells from empty barcodes,^18^^,^^19^^,^^20^ whereas others algorithmically factor out ambient RNA.^12^^,^^21^^,^^22^ Although these post-hoc algorithms use different methods and parameters to attempt to identify contaminant contribution and ambient genes, they are imperfect and often provide an incomplete picture for reliably evaluating data quality. Because of this need, we strive to develop a unique set of metrics to evaluate data quality by specifically considering ambient contamination before any barcode filtering or post-hoc removal of ambient contamination. Rather than mathematically removing ambient RNA, we acknowledge the inability to completely remove contamination algorithmically in every circumstance. Instead, we emphasize the importance of evaluating data quality based on the level of ambient contamination before any data processing to allow end-users to apply appropriate measures to combat the problem. This approach provides an important aspect of quality control that allows researchers to systematically evaluate and report quality metrics of their data, which promotes greater transparency in the upstream design and downstream analysis of scRNA-seq experiments.

In this study, we conducted a series of controlled experiments using an open-source scRNA-seq platform (inDrops) to assess the factors that contribute to ambient RNA contamination. We developed quantitative contamination-focused metrics to assess ambient RNA encapsulated into droplets as reflected in sequencing data. We confirmed the validity of our contamination metrics by applying them to evaluate dissociation protocols known to result in either high cell death or enhanced cell viability. Furthermore, we demonstrated parameters in the microfluidic technology that impact ambient contamination. The significance of quantitative data quality evaluation cannot be overstated in single-cell biology as the accuracy and reliability of downstream analysis are highly dependent on the quality of the input data. We thus provide end-users with quantitative methods to deduce quality from unprocessed data and illuminate how technical parameters behind the technology outside of dissociation protocols can impact contamination.

## Results

### Metrics that focus on ambient contamination can identify poor quality scRNA-seq datasets

We set out to first develop a set of quantitative metrics focused on ambient RNA levels, such that modifications made to downstream protocols can be adequately assessed. For illustrating situations with high and low ambient contamination, we used CellBender^22^ to simulate representative low contamination (ambient UMI count = 100) and high contamination (ambient UMI count = 4000) datasets (see STAR Methods). Other parameters such as number of cells, droplets, and UMI per cell were kept relatively constant between simulations (Figures S1A–S1D). Because ambient contamination is present in cells as well as empty droplets, we developed metrics that apply to unfiltered data. This way, ambient contamination assessment can be performed automatically without the subjectivity of data filtering.

High ambient RNA disrupts the ability to separate real cells from empty droplets, as can be illustrated by the standard UMI count versus log ranked barcodes curve (Figures S1E and S1F), which can also be represented as the cumulative distribution of counts versus ranked barcodes (Figures S1G and S1H). We scaled the total number of barcodes analyzed with respect to the number of expected cells for each dataset to enable comparison between samples with different numbers of encapsulated cells and empty droplets. A sharp change in slope of the cumulative count curve, with a clear inflection point, was observed in the high-quality dataset because real cells contribute to notably larger increments of gene counts than empty droplets when background noise is low. The change in slope was less apparent in the low-quality dataset because ambient genes contribute to high increments of gene counts in empty droplets (Figures S1G and S1H). Although these curves can visually give a gross impression of data quality, we surmise that ambient contamination occurs to various degrees in real datasets. Thus, quantitative metrics are needed to reflect different levels of contamination.

**Figure 1:**
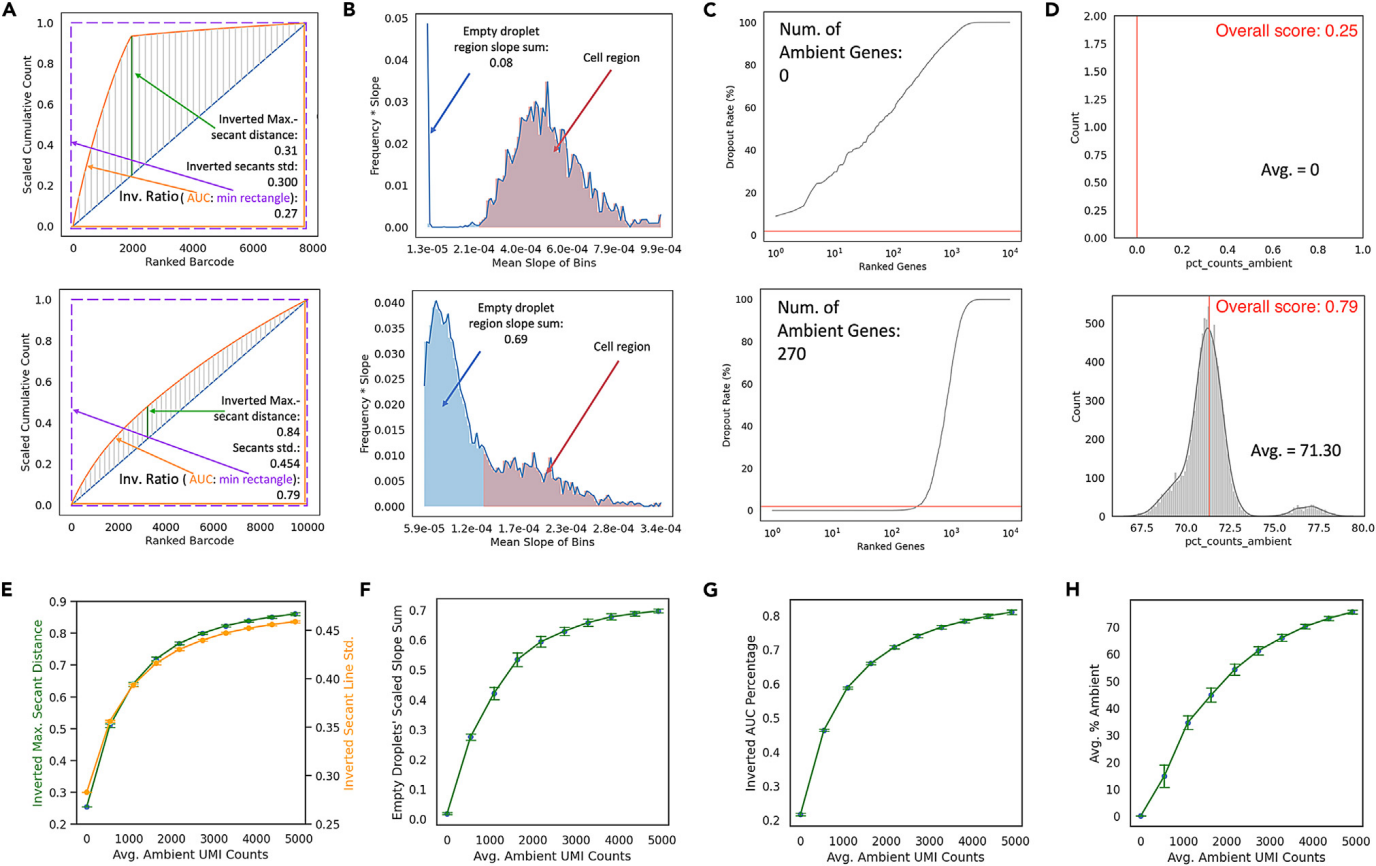
Ambient contamination metrics robustly reflect data quality on simulated datasets (A) Scaled cumulative total transcript counts over ranked barcodes by total transcript counts for datasets simulated with (top) low ambient level and (bottom) high ambient level. Secant lines from the curve to the diagonal line are colored in gray with the line with maximal secant line colored in green, which were used to calculate inverted maximal secant distance and secant line standard deviation. The area under curve (colored in orange) and the minimal rectangle circumscribing (dashed purple line) were used to calculate the inverted AUC percentage. (B) Scaled representation of the slope distribution histograms shown in Figures S1I and S1J for (top) low and (bottom) high ambient datasets shown in A. The x axis values are midpoint of each bin in the slope distribution histogram, and the y axis values are multiplication product of the bin midpoint values and the bin heights. The region representing slopes that were below the threshold were considered as empty droplets and were colored in blue. The sum of these datapoints is quantified as empty droplets' scaled slope sum. (C) Distribution of dropout rate of genes ranked by ascending dropout rate for datasets simulated with (top) low and (bottom) high ambient level. The pink line is drawn at 2% dropout rate, the cut-off below which a gene will be defined as ambient. (D) Distribution of percentage of ambient genes expressed per cell for dataset simulated with (top) low and (bottom) high ambient level. The mean percentage is quantified. The AmbiQuant overall score is labeled in red. (E–H) (E) Maximal secant distance (green) and secant line standard deviation (yellow), (F) AUC percentage, (G) cell’s scaled slope sum, and (H) percent counts ambient over different ambient levels for simulations. Line plots shown as mean ± stdev of n = 1000 replicates for each ambient level.

The ability to separate true signals from background contamination can be assessed in two ways: geometrically or statistically. Geometrically, a cumulative count curve resembling a rectangular hyperbola reflects this sharp change in slope, and hence higher quality, while the resemblance to a straight line reflects the opposite. For quantification, we defined secant lines connecting each point on the cumulative count curve to the diagonal line linking the origin to the last data point of the cumulative count curve (Figure 1A). The high-quality dataset, because of its resemblance to a rectangular hyperbola, has a larger maximal secant line distance as well as a larger standard deviation over all secant line distances, as compared to the low-quality dataset (Figure 1A). We also assessed the direct resemblance of the curve to a rectangle by calculating an area ratio between the area under the cumulative count curve and the minimal rectangle circumscribing the curve, which we termed AUC percentage over minimal rectangle, with high quality data occupying more of the rectangular area (Figure 1A). We then inverted these quantitative assessments to establish contamination metrics, such that they scale in proportion to the degree of contamination according to the geometry of the cumulative counts versus ranked barcodes curve (Figure 1A).

We also used statistical distributions to quantify ambient contamination, by first generating a distribution of slopes at each point of the cumulative count curve displayed as a histogram, where bin widths are the range of slopes and bin heights are the number of data points with a slope falling into the range of the bin width (Figures S1I and S1J). Taking the midpoint of each bin and multiplying the midpoints value with its bin height, we generated a line plot with the midpoints being x values and the product of multiplication as y values, which is a scaled representation of the slope distribution that has a higher density over data points that have higher slope values. Because high slope data are hypothetically contributed by real cell’s transcript counts, the scaling achieves an increased contribution of real cells, weighted by their transcript count, against the contribution of empty droplets (Figure 1B). The scaled distributions were normalized to one to enable cross dataset comparisons. We surmised that a contaminated dataset should have a slope distribution closer to unimodal, because of indistinguishable cells and empty droplets, while a high-quality dataset should have a multimodal slope distribution. Thus, a cut-off was determined to separate an “empty droplet” slope distribution from a “cell” slope distribution. This cut off was determined to be one standard deviation above the median of all slopes to approximate the “empty droplet” distribution, because most barcodes are empty droplets in a scRNA-seq experiment (Figure 1B). The sum of scaled slopes below this threshold, denoting data points that are potentially background ambient signals, is a quantitative metric that scales with the dataset’s contamination level (Figure 1B). Aside from slope distributions, we further constructed distributions characterizing ambient genes. These are genes detected to be present in most barcodes (both cells and empty droplets) and have a dropout rate of less than 2% (Figure 1C) (see STAR Methods). The number of ambient genes and the mean percentage of the ambient gene expressed per cell quantitatively differed between high- and low-quality datasets (Figures 1C and 1D). Thus, summary metrics from various statistical distributions can also be used to quantitatively assess ambient contamination.

To verify the robustness of these metrics to quantitatively assess ambient contamination, we evaluated simulated datasets at 10 ambient levels over n = 1000 replicates. The contamination metrics - inverted maximal secant distance, inverted secant line standard deviation, inverted AUC percentage, sum of weighted slopes under threshold, average percentage of ambient genes, and the number of ambient genes all quantitatively increased in proportion to the ambient level set (Figures 1E–1H and S1K). We also used real datasets to simulate different sequencing depths and ambient contamination levels and showed that our metrics are resistant to sequencing depth variations but are specific to changes in ambient contamination levels (Figures S1L–S1O). Meanwhile, ambient count percentage and ambient gene counts are sensitive to different sequencing depths and, thus, these metrics need to be used with more scrutiny (Figures S2P–S2R). Together, these results demonstrate that the quantitative metrics derived, which we termed contamination metrics, can robustly inform scRNA-seq data quality on a continuous scale based on ambient RNA contamination. For simplicity, we developed an overall score combining several of the contamination metrics, bound between ‘0’ (best quality – perfect signal-to-noise ratio) to ‘1’ (worst quality – all noise). The approach we took to quantify ambient contamination is implemented in a package called AmbiQuant (https://github.com/Ken-Lau-Lab/AmbiQuant.git).

### Application of contamination metrics revealed ambient contamination in datasets that passed standard QC

We first compared the performance of the contamination metrics against standard metrics as defined by Hong et al. on inDrops scRNA-seq datasets (Hong et al., 2022^17^). These metrics are prevalently used in current scRNA-seq QC, and include total number of cells, average percent mitochondrial gene expression, average total transcripts per cell, and average total number of genes detected per cell. Note that the total number of cells is not the cells that enter the chip to be encapsulated, because that number is standardized by visualizing and counting the rate of cell entry into the cell hopper of the microfluidics chip. Instead, the total number of cells represents the number of cells identified in the data after barcode filtering downstream, providing a glimpse of the actual numbers of intact cells that survived encapsulation.

**Figure 2:**
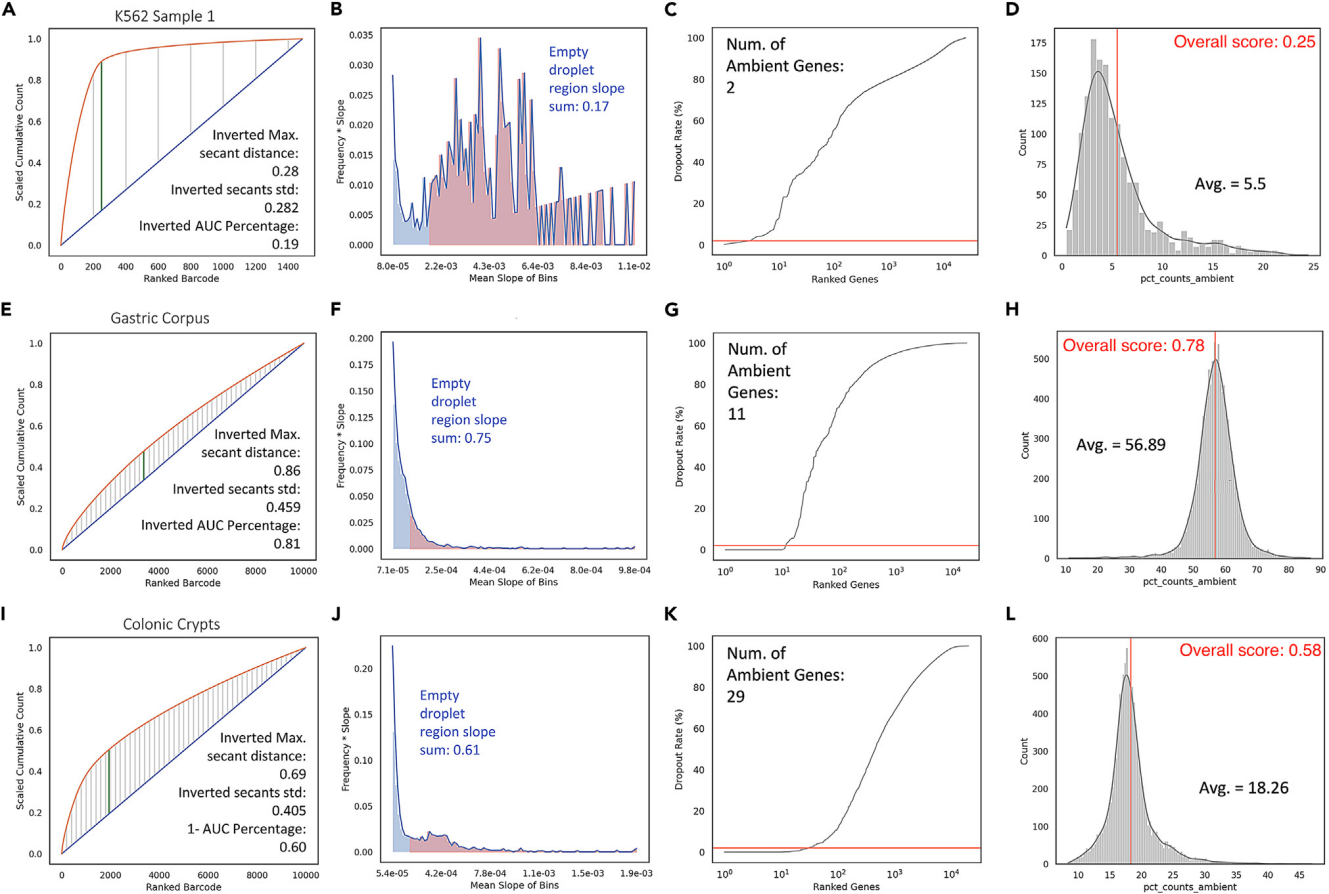
Contamination metrics on experimental datasets inform data quality on a continuous scale Ambient contamination plots and metrics, formatted similarly to Figure 1 of experimental datasets of different quality: (A–D) K562 (Sample 1) cell line, (E–H) mouse gastric corpus, (I–L) and mouse colonic crypts.

We applied contamination metrics on datasets generated from the inDrops platform without any modification (standard inDrops).^9^ K562 cells, as optimized in the original inDrops manuscript, demonstrated low contamination based on our metrics (AmbiQuant Overall Score: Sample 1–0.25; Sample 2–0.31; Sample 3–0.27), as well as standard QC metrics such as mitochondrial count percentage per cell (Figures 2A–2D and S2A–S2K and Table S1). Cultured cells maintain very high viability after minimal or no dissociation, leading to high data quality. In contrast, we also dissociated gastric corpus tissues in an unoptimized fashion and applied inDrops (see STAR Methods). The gastric corpus is the site of stomach acid production and houses various types of gastric cells, including acid-producing parietal cells.^23^ Thus, dissociated single cells in this environment are exposed to extrinsic stress and damage. Standard data analysis revealed obvious QC failure in scRNA-seq data generated, as reflected by a high mitochondrial percentage, low number of genes detected, and general inability to detect known cell types (Figure S2L and Table S1). Poor data quality was also captured by our contamination metrics (AmbiQuant Overall Score: 0.78) (Figures 2E–2H and Table S1). Although obvious QC failure is easy to detect, there are intermediate cases where low data quality can be concealed within data that qualitatively passed QC, such as the case with the colonic epithelium. The colonic epithelium consists of a unilaminar layer of connected differentiated and undifferentiated epithelial cells. Differentiated cells do not self-renew and undergo anoikis when dissociated from their neighbors, increasing the propensity of dying cells in suspension.^15^ More importantly, secretory cells, including goblet cells, are constantly under endoplasmic reticulum stress (because of heightened protein production) and are packed with tubulovesicular elements for protein secretion, leading to increased fragility.^24^ Standard inDrops scRNA-seq of colonic epithelium^25^ did not lead to QC failure, and two major lineages of secretory and absorptive cells can clearly be delineated from the data (Figure S2M and Table S1). However, closer examination of the data revealed that the high expressing, Goblet cell-specific gene *Muc2* was found in every cell, demonstrating a high degree of ambient contamination (Figure S2N). Standard quality metrics such as mitochondrial count percentage, total UMI count, and total genes were unable to distinguish datasets of low versus high quality arising from ambient RNA, but our contamination-based metrics could (AmbiQuant Overall Score: 0.58) (Figures 2I–2L and Table S1). These results demonstrate that our contamination metrics can reveal previously missed problems of ambient RNA in data generated using droplet-based scRNA-seq.

### Pre-encapsulation variables affect scRNA-seq data quality and cell type diversity

**Figure 3:**
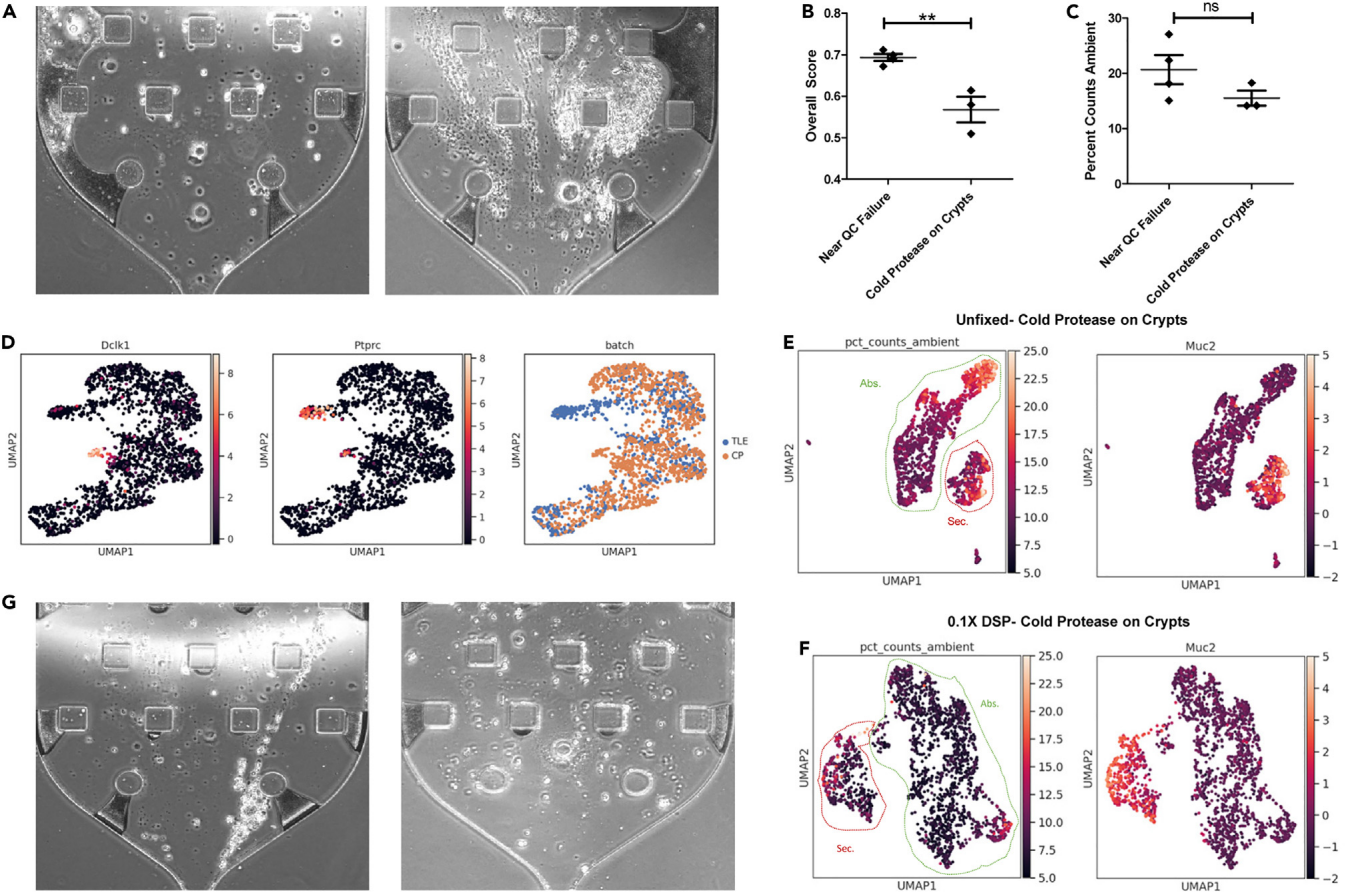
Pre-encapsulation variables affect scRNA-seq data quality and cell type diversity (A) Live hopper visualization of (left) viable single cells and (right) dying cells. (B and C) Quantification of (B) AmbiQuant overall score, (C) percent counts ambient comparing near QC failure runs (MACs enzyme on minced tissue, cold protease on minced tissue, MACs enzyme on minced, and Collagenase/DNase on Crypts) and cold protease dissociation on crypts. Mean with SEM as error bars for n = 3 or 4 samples. ∗∗p < 0.01 by t-test. (D) UMAP embedding of filtered cells from (blue) TrypLE and (orange) cold protease datasets. Expression of *Dclk1*, a tuft cell marker, and *Ptprc*, an immune cell marker, were overlaid. (E and F) UMAP overlay with percent counts ambient or *Muc2* expression for (E) unfixed cells or (F) fixed cells prepared with cold protease dissociation on crypts. Secretory (red) and absorptive (green) lineages are outlined. Gene expression values on scale bars are Z-scores of normalized values described in STAR Methods. (G) Live hopper visualization of (left) unfixed cells and (right) cells fixed with 0.1 X DSP.

Using colonic epithelium as our model system, we further tested our contamination metrics against pre-encapsulation variables that were shown to impact downstream ambient contamination in a tissue-specific manner.^14^ We first compared scRNA-seq data quality following the standard HTAPP tissue dissociation protocol (https://doi.org/10.17504/protocols.io.busfnwbn) compared with our standard crypt chelation strategy followed by cold protease dissociation (cold protease and DNase cocktail; see STAR Methods). The HTAPP protocol that requires the act of mechanical separation via total tissue mincing followed by a 20-min enzymatic dissociation at 37°C led to cell death and damage, which can be visualized in the cell hopper of the microfluidic encapsulation chip as streaks of cellular material as opposed to intact cells (Figure 3A). Quantitatively, tissue mincing, followed by either warm or cold dissociation, led to poor data quality as reflected by both standard and contamination metrics (Table 1; Figures S3A and S3B). The inability to distinguish different cell populations or cells from empty droplets in these datasets is similar to the QC failure of the gastric dataset above, and thus, they are labeled as near QC failure results. To isolate single-cell dissociation steps, we eliminated mechanical tissue separation and tested different dissociation enzymes only on isolated crypts that were produced by chelation (see STAR Methods). Standard dissociation enzymes such as the collagenase/DNase cocktail, and the Miltenyi MACs enzyme also led to poor contamination metrics when applied to crypts and resulted in near QC failure (Table 1, Figures S3C and S3D). These dissociation enzymes require long incubation times at 37°C which may accelerate biological processes including cell death in single-cell suspensions, as opposed to cold protease which has been shown to preserve viability by the opposite effect.^26^ Contamination metrics calculated on cold protease dissociation on crypts datasets were quantitatively lower than the four near-QC failure datasets generated by mechanical mincing and/or warm enzymatic dissociation (Figures 3B and S3E–S3H, Table 1). The percent count ambient metric was more variable because the identities of ambient genes differed amongst techniques, but trended lower for cold protease dissociation on crypts, as expected for high quality datasets (Figure 3C). Standard QC metrics, such as number of genes and transcripts detected, also demonstrated higher data quality derived from cold protease dissociation on crypts compared to conditions that led to near QC failure (Figures S3I–S3L and Table 1). It is also well-known that different single-cell dissociation strategies can lead to different cell type representation.^14^ We show here through integrated UMAP and clustering analysis that cold protease dissociation recovered more tuft cells, while more immune cells were recovered by short term TrypLE dissociation of crypts (Figure 3D). Standard dissociation strategies can result in acceptable data quality with hardy cell types such as cancer cells; however, normal cells are more sensitive to stressors at the tissue and cell level during handling that can affect downstream ambient contamination. Our results confirm the improvement of scRNA-seq data quality using cold protease dissociation by Adam et al.^26^ and demonstrate that contamination-focused metrics can explain how known pre-encapsulation factors impact data quality.

**Table 1:** Quality control metrics to assess the impact of pre-encapsulation protocols

	MACS Enzyme on Minced Tissue	Cold Protease on Minced Tissue	MACS Enzyme on Crypts	Collagenase and DNase on Crypts	Cold Protease on Crypts	0.1X DSP	1% PFA
**Contamination metrics**							

Empty Droplet Scaled Slope Sum	0.82	0.78	0.84	0.80	0.62	0.53	0.67
Inverted Max Secant Distance	0.78	0.83	0.86	0.79	0.68	0.65	0.67
Inverted Secant Line St.Dev.	0.44	0.45	0.46	0.44	0.40	0.40	0.40
Inverted AUC Percentage	0.70	0.76	0.80	0.72	0.58	0.54	0.56
Avg. Percent Counts Ambient	27.06	22.34	15.06	18.14	15.52	10.48	7.11

**Standard metrics**							

Total Number of Cells	730.00	295.00	349.00	249.00	1019.67	2049.00	313.00
Avg. Percent Counts Mitochondria	9.86	5.06	3.18	5.83	4.03	2.05	0.57
Avg.Total Genes per Cell	990.96	1434.31	1989.21	2373.82	3682.52	3201.20	1685.39
Avg. Total Transcripts per Cell	1860.70	3220.45	4281.85	8155.20	12509.90	8209.73	4308.89

**AmbiQuant**							

Overall score	0.69	0.70	0.71	0.67	0.57	0.52	0.56

Because cold protease dissociation on isolated crypts resulted in the optimal balance between cell recovery and ambient contamination, we then assessed whether fixation immediately after dissociation would further improve data quality. We surmised that fixation would contain and trap all RNA within a cell and thus, will prevent ambient RNA from leaking into the surrounding buffer. We assessed two fixation strategies previously employed in scRNA-seq studies, 1% light PFA fixation used previous in combinatorial indexing^6^ and 0.1X dithiobis(succinimidyl propionate) (DSP), a reversible crosslinker commonly used in pulldown studies.^27^ Cells fixed with 1% PFA led to QC failure, mainly because of fixation-induced degradation of RNA that disrupted library preparation (Figure S3M). However, cells fixed with 0.1X DSP led to lower contamination metrics compared to fresh tissues, again with clear delineation between secretory and absorptive cells with less ambient transcripts and non-specific *Muc2* (Figures 3E and 3F and Table 1). These results show that fixation can indeed contain RNA within cells and decrease ambient contamination arising from cell death during dissociation which can also be visualized in the cell hopper (Figure 3G).

### Microfluidic manipulations can affect cell death and subsequent ambient contamination in downstream data

**Figure 4:**
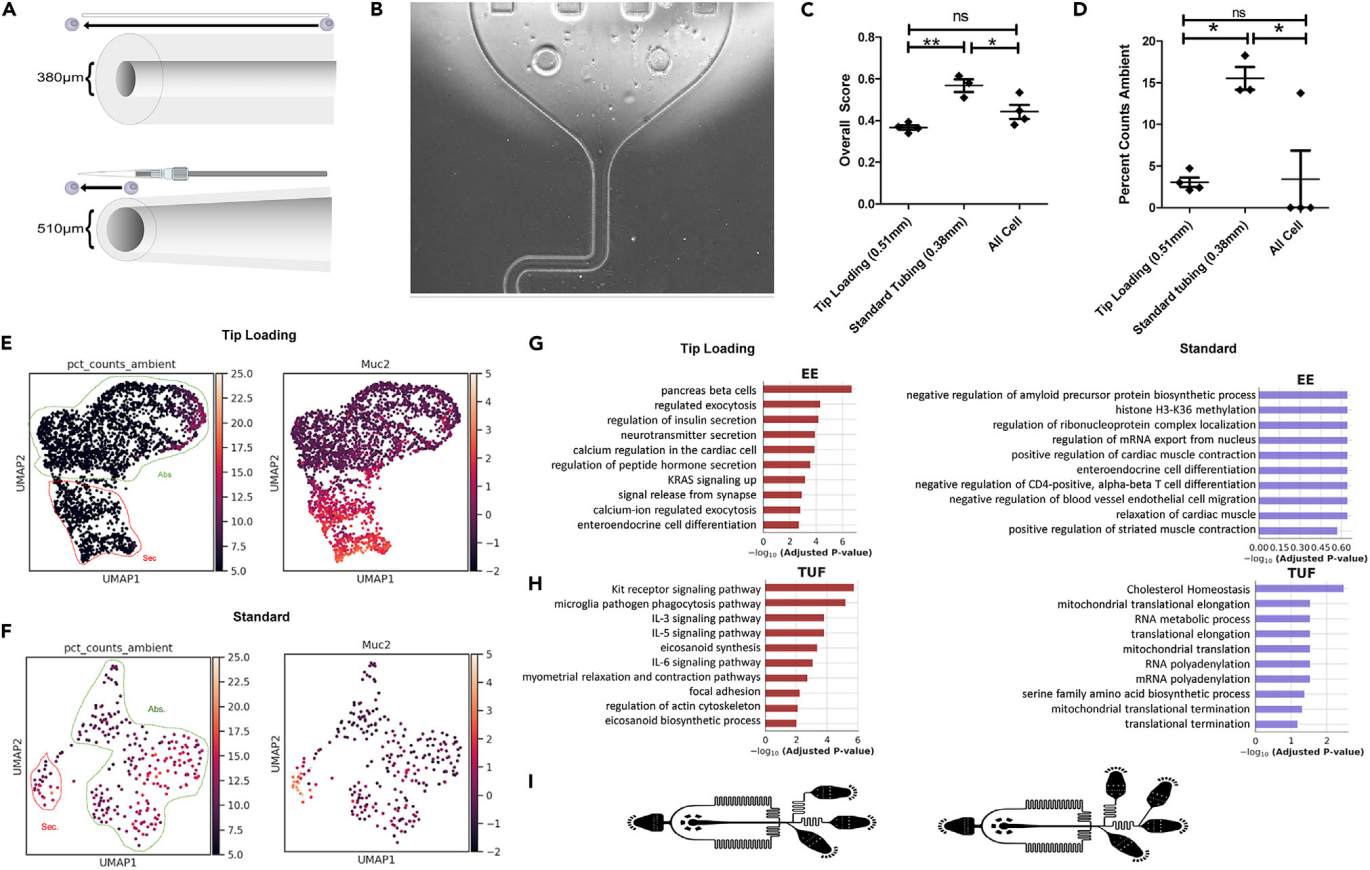
Microfluidic manipulations can affect cell death and subsequent ambient contamination in downstream data (A) Schematic of standard loading (top) and tip loading (bottom). (B) Live hopper visualization of viable single cells from tip loading apparatus. (C and D) Quantification of (C) AmbiQuant overall score, (D) percent counts ambient comparing various microfluidics manipulations. Mean with SEM as error bars for n = 3 or 4 samples. ∗p < 0.05, ∗∗p < 0.01 by ANOVA followed Tukey post-test. (E and F) UMAP overlay with percent counts ambient or *Muc2* expression for (E) tip loading or (F) standard loading. Secretory (red) and absorptive (green) lineages are outlined. Gene expression values on scale bars are Z-scores of normalized values described in STAR Methods. (G and H) Comparison of functional enrichment analysis datasets derived from tip loading (higher data quality) and standard loading (lower data quality) looking at (G) enteroendocrine (EE) and (H) Tuft (TUF) cells. (I) Schematic for standard inDrops chip (left), and All Cell chip (right).

The reduction of ambient contamination by post-dissociation fixation suggests that cells undergo continuous cell death after tissue handling. We also used live/dead cell sorting to maximize cell viability before cell encapsulation, which surprisingly did not improve downstream data quality (Figures S4A–S4D). The standard inDrops system loads cells into the encapsulation junction using tubing that are 0.38 mm in diameter fed by a syringe pump. Thus, we next assessed whether cell traveling through tubing affects downstream ambient contamination. No significant difference in cell viability was observed between cells that traveled through 20 cm of the 0.38mm tubing and directly released from the syringe pump (Figure S4E), indicating that off-chip time does not play a critical role. However, significantly improved results were obtained when a custom alternative cell loading setup was used to load cells directly into the encapsulation chip. This setup uses a fabricated pipette tip/syringe hybrid loading system with a minimum 0.51 mm diameter (tip loading) to reduce cell travel time in narrow microfluidic tubing (Figure 4A). The modification led to more viable cells as visualized in the cell hopper (Figure 4B), as well as significantly and consistently reduced contamination and increased number of encapsulated cells (Figures 4C, 4D and S4F–S4N and Table 2). Goblet cells were also more consistently recovered compared with standard loading (Figures 4E and 4F). Application of tip loading was also able to improve data quality on colonic samples prepared with minced tissue in place of crypt isolation (Table S2). Thus, we pinpointed that the major contributor of ambient contamination and poor data quality in droplet-based scRNA-seq is a combination of traveling through narrow tubing followed by microfluidic encapsulation.

**Table 2:** Quality control metrics to assess the impact of post-dissociation parameters

	0.38mm Standard Tubing	0.51 mm Tip Loading	4:1 All Cell Chip Dilution	≥1:1 All Cell Chip Dilution
**Contamination metrics**				

Empty Droplet Scaled Slope Sum	0.62	0.31	0.82	0.56
Inverted Max Secant Distance	0.68	0.46	0.83	0.56
Inverted Secant Line St.Dev.	0.40	0.34	0.45	0.36
Inverted AUC Percentage	0.58	0.34	0.76	0.42
Avg. Percent Counts Ambient	15.52	3.07	19.24	4.59

**Standard metrics**				

Total Number of Cells	1019.67	2880.75	156.00	376.67
Avg. Percent Counts Mitochondria	4.03	4.25	3.54	4.06
Avg.Total Genes per Cell	3682.52	2803.87	1857.88	2475.67
Avg. Total Transcripts per Cell	12509.90	7702.99	5408.91	8095.85

**AmbiQuant**				

Overall score	0.57	0.36	0.70	0.46

To further demonstrate the differences in biological interpretation between higher quality versus lower quality data, we performed functional enrichment analysis on cell populations identified in scRNA-seq datasets generated from tip loading (higher quality) compared to standard (lower quality). The functional terms enriched for secretory Goblet cells and absorptive colonocytes were similar between high- and low-quality datasets. Goblet cells were enriched for O-linked glycosylation and unfolded protein response functions, both of which are required for the production and secretion of mucins (Figures S4O and S4P).^28^^,^^29^ Colonocytes were enriched for functions fatty acid metabolism and monocarboxylic acid transport, both key functions for reabsorption (Figures S4O and S4P).^30^ These cells were also enriched for hypoxia and apoptosis, being that they are located at the luminal surface in contact with the anaerobic environment.^31^ Both high- and low-quality datasets were able to decipher the canonical functions of these cell types because they contribute most to ambient contamination (goblet cells being sensitive to cell death and colonocytes being most abundant) and, thus, their transcriptomes are not masked. However, functional enrichment in other cell types showed stark differences between high- and low-quality datasets. For instance, MYC and E2F transcriptional targets downstream of WNT signaling in colonic stem cells are detected in the high-quality dataset but not detected in the low-quality dataset (Figures S4O and S4P).^32^ More strikingly, enteroendocrine cells, enriched for their canonical functions of hormone and neurotransmitter secretion,^33^ and tuft cells, enriched for eicosanoid synthesis, immune signaling, and cytoskeletal structure, were completely mischaracterized in low quality datasets (Figures 4G and 4H).^34^^,^^35^^,^^36^ These results demonstrate how ambient contamination can adversely affect downstream functional analysis of scRNA-seq data, especially for cell types that do not contribute to the contamination.

We also hypothesized that ambient contamination can be reduced post-cell death via microfluidic manipulations. We utilized an alternative chip design (“All Cell”) that included another reservoir and inlet for dilution buffer, with the idea that ambient RNA in the loading buffer can be diluted out immediately before cells are encapsulated into droplets (Figure 4I). Loading the suspension in a ratio of 4:1 (cell: dilution buffer) did not improve data quality. Increasing the dilution ratio to ∼1:1 resulted in lower contamination metrics (Figures 4C and S4F–S4N and Table 2). However, because of dilution of the cell suspension before encapsulation, the number of cells recovered was decreased (Figure S4K). These results demonstrate that microfluidics manipulation can improve data quality pre- and post-encapsulation by altering cell exposures and diluting out ambient contamination, respectively. However, various tradeoffs, for instance, the number of cells recovered, need to be acknowledged.

### Application of contamination-focused metrics to evaluate single-cell/single-nuclei dissociation protocols on different tissues

**Figure 5:**
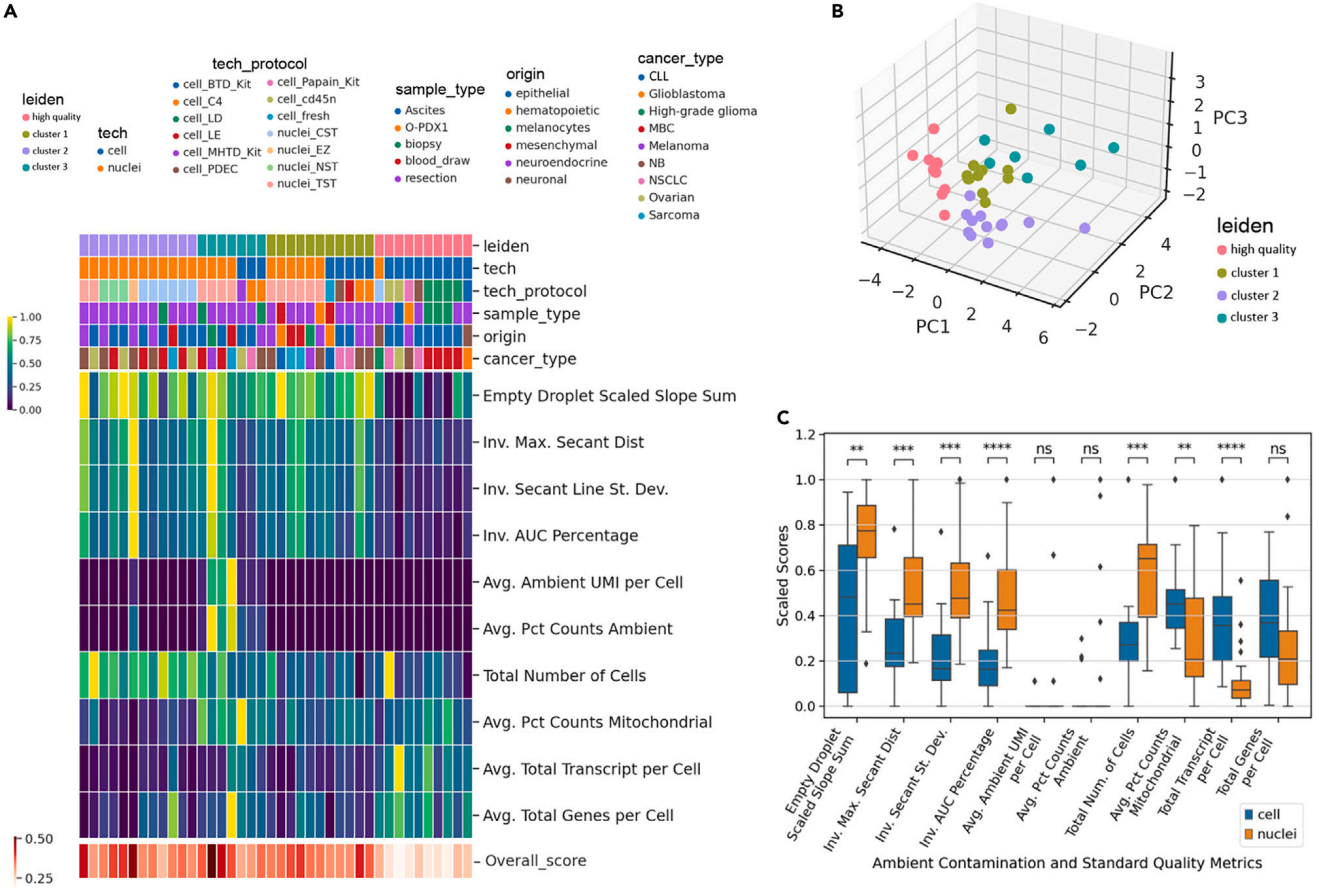
Ambient contamination and quality control metrics reveal impact of intrinsic and extrinsic factors on data quality (A) Heatmap of ambient contamination and standard QC metric scores with HTAPP datasets as columns grouped by Leiden clusters. Metrics are shown as rows, where the first ten rows are individual metrics, whose colors correspond to the top left color bar. The last row is the AmbiQuant overall score for the ambient contamination metrics, colored in red corresponding to the bottom left color bar. The Leiden cluster labels and labels of isolation technique, technique and protocol combination, sample type, tissue origin, and cancer type are shown as color bars above the heatmap. Metric scores are normalized between 0 and 1 for each row for visualization. Abbreviations - cell: scRNA-seq; nuclei: snRNA-seq; BTD: brain tumor dissociation; C4: collagenase 4 and DNase I; LD: Liberase TM and DNase I; LE: Liberase TM, elastase and DNase I; Miltenyi Biotec human tumor dissociation; PDEC: pronase, dispase, elastase, collagenases A and 4 and DNase I; Paipan: (cysteine protease); cd45n: CD45^+^ depletion; CST: CHAPS with salts and Tris; EZ: EZPrep; NST: Nonidet P40 with salts and Tris; TST: Tween with salts and Tris; O-PDX1: orthotopic patient-derived xenograft; CLL: Chronic lymphocytic leukemia; MBC: Metastatic breast cancer; NB: Neuroblastoma; NSCLC: Non-small cell lung carcinoma. (B) Three-dimensional scatterplot of the first 3 principal components of the ambient contamination and standard QC metric score matrix colored by Leiden cluster labels. (C) Boxplot comparing the metric scores between single-cell and single-nucleus sequenced samples. Two-sided Mann-Whitney-Wilcoxon test performed between single-cell and single-nuclei groups. ∗∗p < 0.01, ∗∗∗p < 0.001,∗∗∗∗p < 0.0001.

Although there is anecdotal knowledge in the field about the performance of sc-/sn-RNA-seq on various tissues with different dissociation protocols, downstream data quality has not been evaluated in a comprehensive manner. Slyper et al. presented a systematic evaluation of various dissociation protocols on 8 cancer types, generating 40 sc-/sn-RNA-seq datasets.^14^ These datasets were generated within the same laboratory, which minimized some systematic variations, but their performance has only been evaluated using standard QC (such as number of genes/UMI per cell, cell type composition, etc.). We visualized standard and contamination metrics applied to the 40 datasets as a clustered heatmap and observed that different metrics map onto different types (clusters) of datasets (Figure 5A). Although some covariation was observed for some sets of metrics (for instance, Inv. Max.Secant Dist/Inv. Secant Line St. Dev./Inv. AUC Percentage and Avg. Ambient UMI per cell/Avg. Pct Counts Ambient), other metrics are distinct. For metrics that covary, the correlations were not perfect 1-to-1 correlations, indicating that they capture slightly different information regarding data quality (Figures S5A–S5C). To leverage the orthogonal, non-covarying portions of the metrics to describe data quality, we performed principal component analysis (PCA) to reduce dimensions followed by clustering and visualization in UMAP space (Figures 5A, 5B, and S5D). Samples organized into four clusters based on PCA-reduced metrics space, with one high-quality and three lower-quality clusters. The three lower-quality clusters addressed different aspects of data quality: cluster 1 possessed a decreased number of captured cells/nuclei, whereas cluster 3 showed more ambient gene contribution (Figures 5A, 5B, and S5E–S5M). Cluster 2 displayed a high number of identified cells/nuclei, even though both contamination metrics and standard metrics indicated lower data quality. We then examined different sample types and preparation conditions to elucidate factors that contribute to different data quality in each cluster (Figures 5A and S5N–S5R). The high-quality cluster was almost entirely made of single-cell samples, whereas single-nucleus samples were abundant in the lower-quality clusters. (Figures 5A and S5O). This observation was further supported by plotting the different metrics between single-cell versus single-nucleus datasets (Figure 5C). The contamination metrics were universally higher for single-nucleus samples compared with single-cell samples, while the standard metrics were more variable. While the number of ambient genes and the percent ambient genes were more variable as explained previously, they trended unfavorably for snRNA-seq. The increased number of nuclei/cells captured by snRNA-seq compared to scRNA-seq can be attributed to a harsher and more comprehensive nuclei isolation strategy tolerated by this approach, while mitochondrial percentages were lower, being that nuclei do not contain mitochondria. Total transcript counts of snRNA-seq datasets were significantly lower than single-cell samples, because the cytoplasm was absent in such preparations. While the standard metrics are variable, which can be attributed to various experimental factors outside of data quality (e.g., real depth, transcriptome size), our contamination metrics reliably indicated that single-nucleus specimens have lower data quality than single-cell specimens. Single-nucleus isolation procedures generate nuclei containing adhered ribosomes and RNA that can easily shed from the majority of nuclei into the loading buffer to create ambient contamination.

Further inspection indicated that certain techniques such as single-cell Liberase and DNase (LD) and CD45^+^ depletion (CD45n) were consistently yielding higher-quality data, whereas other protocols such as single-cell C4 (Collagenase 4 and DNase I) resulted in more contaminated datasets (Figures 5A and S5N). There were also differences in the buffers used in snRNA-seq approaches. For example, Tween with salts and Tris (TST) was in cluster 1 and 3 with more mitochondrial and ambient contribution, whereas Nonidet P40 and CHAPS with salts and Tris (NST and CST) generally presented in cluster 2 with higher number of nuclei identified. However, cancer type, tissue origin, or tissue collection procedures scattered randomly amongst clusters, indicating no concordance with data quality (Figures 5A and S5P–S5R). These results indicate that the cell/nuclei preparation protocol was the major factor impacting data quality.

## Discussion

Droplet-based scRNA-seq technology has become widely used for understanding tissue biology and heterogeneity. Large consortia have been established for human tissue profiling using scRNA-seq as a central approach.^37^^,^^38^ However, several technical artifacts, such as ambient contamination, have not received sufficient attention. The contamination problem is particularly problematic for healthy tissues compared to cancer tissues, as differentiated cells, especially fragile secretory cells, are more prone to cell death. The most prevalent mode for quality control of a scRNA-seq dataset is the ability to distinguish cell populations via marker genes in some reduced dimension space. Other standard metrics such as feature counts, transcript counts, and cells recovered in the data do not directly reveal ambient contamination. Although canonical cell types may still be identified by highly expressed marker genes in a dataset heavily contaminated by ambient RNA, downstream analysis regarding gene programs, states, and pathways will be significantly confounded. For instance, “mixed lineage” cells may just be artifacts of ambient contamination and not a genuine biological phenomenon. We also speculate that a large portion of batch effect may arise from ambient contamination whose composition is random for every set of experiment. Furthermore, ambient contamination may even prevent cells from being effectively distinguished from empty droplets, resulting in significant manual efforts in data filtering. Given the widespread use of scRNA-seq, we believe that end users of publicly available data will benefit from having tools to assess data quality before their use. Quality control of scRNA-seq experiments is essential for maintaining data integrity, optimizing experimental workflows, filtering out poor-quality data points, ensuring comparability and reproducibility across datasets, and downstream analysis and biological interpretation. We provide quantitative metrics using post-alignment counts data before filtering that reveal ambient contamination in scRNA-seq data. Our contamination metrics essentially capture the ability to identify signal (biological transcripts) from noise (ambient transcripts) within a scRNA-seq experiment. AmbiQuant metrics can be easily applied across large cohorts of data to assess overall quality of each dataset. Furthermore, these metrics augment current ambient decontamination algorithms to provide a more accurate picture of data quality. While we acknowledge that investigators already perform substantial filtering on their datasets, we would like to highlight two important improvements enabled by our metrics. First, conducting quality control and filtering on a per-dataset basis can introduce dataset-specific bias because of the labor-intensive nature of the process. The variations in QC practices result from human involvement. Second, we emphasize the automatic generation of these metrics at the cohort level rather than individual datasets. This approach allows for the efficient identification of relatively low-quality datasets without the need for extensive filtering efforts. Aside from ambient contamination that contributes to noise, our metrics can also identify experiments with excessively low transcript counts that approach baseline signals. For instance, the same level of contamination may more adversely affect cells with inherently small transcriptomes (e.g., lymphocytes) or single-nuclei runs where cytoplasmic signals are largely excluded, as compared to cells with large transcriptomes and high signals (e.g., epithelial cells). However, we found that our metrics are resistant to variation in sequencing depths, whereas common metrics such as total UMI and ambient gene counts are not. This is because of the proportional scaling of both signal reads and contamination reads with number of total reads. Cell number can affect data quality in a different manner to ambient contamination if the starting material becomes so minute that the ability to amplify cDNA and prepare sequencing-amenable libraries is affected. This would affect the number of genes and transcripts detected stemming from reduced diversity and increased duplication level of the sequencing library.

Several computational packages have been developed to address ambient contamination post-hoc. EmptyDrops, dropkick, and DropletQC leverages the ambient contamination profile for automatically identifying cells from empty droplets.^18^^,^^19^^,^^20^ DecontX, SoupX, and CellBender are tools for factoring out the contribution of ambient RNA in the resultant counts data matrix.^12^^,^^21^^,^^22^ However, it cannot be assumed that post-processing algorithms can be relied on to turn all poor-quality data to high-quality data, because algorithms are built on certain assumptions that may not hold true in all experimental conditions. Therefore, it is difficult to predict when and where these algorithms will fail (Figures S5S and S5T). Thus, we surmise that addressing the problem experimentally on the front end will be the best strategy. We validated our contamination metrics to show the well-known factor that cell death encountered in mechanical and standard enzymatic dissociation results in ambient contamination. Leaked DNA/chromatin from dying cells in the microfluidic encapsulation chip can lead to a cascade of cell death events, as nucleic acid material can act as danger signals for TLR9 and cGAS.^39^ However, we also identified factors downstream of tissue handling that contribute to ambient contamination. We have identified that the primary cause of this contamination is the traversal of cells through narrow tubing in combination with microfluidic encapsulation. We speculate two explanations for this phenomenon. First, traversal of cells in this fluidic setup may induce an alternative cell state that sensitizes cells to death subsequently during the encapsulation process. Second, the interaction between the cells and the tubing results in adhered cells and subsequent DNA/chromatin release that promotes further cell death during the duration of encapsulation. The degree of contamination also varies by protocol, with single nuclei sequencing possessing more severe ambient contamination. Contamination can be partially resolved by fluidic manipulations that either reduces cell death or dilutes out the contamination. Although microfluidics parameters cannot be easily altered in commercial systems, there is still significant value in exploring and documenting these factors in affecting data quality. It is worth noting that there are now alternative droplet-forming technologies, each employing distinct methods for cell encapsulation. Developing an understanding of how these factors influence data quality can empower users to make well-informed decisions regarding platform selection. These factors can also help improve performance of current commercial systems.

A common misconception in the field is the efficacy of snRNA-seq in dealing with cell death and ambient RNA. Nuclei are thought to be stiffer, smaller in size, and more resistant to mechanical damage. However, resistance to death and ambient contamination are not necessarily related. For instance, attached RNA, which can be sheared off and released into the buffer, is a potential source of contamination independent of the integrity of the nuclei. There is support for this adhered RNA in the literature, as the nuclei-attached ribosomes can be visualized^14^ and snRNA-seq data have been used for RNA Velocity that utilizes spliced RNAs that are cytoplasmic but attached to nuclei.^40^ Although snRNA-seq makes single-cell analysis possible for frozen and difficult-to-dissociate tissues,^41^ end users should balance these factors when deciding whether to use cells or nuclei for their single-cell analysis.

### Limitations of the study

Our metrics do not capture single-cell or cell population-specific data quality. Instead, our methods focus on evaluating the overall data quality for each dataset. For instance, if rare cell populations are lysed but do not contribute significantly to ambient contamination, our metrics will not identify those events. Local events of contamination, such as a cell that lyses and immediately contributes ambient RNA to its neighboring cell, will also not be identified if occurring at small scales. While systematic, our study did not exhaustively test every technical factor that can affect data quality.

## STAR★Methods

### Key resources table

**Table:** 

REAGENT or RESOURCES	SOURCE	IDENTIFIER
**Chemicals, peptides, and recombinant proteins**		

Ethylenediaminetetraacetic acid (EDTA) 0.5M	Corning	46-034-CI
Dithiothreitol (DTT) 1M	Teknova	D9750
Deoxyribonuclease I (Dnase I)	Sigma-Aldrich	DN25
Protease from Bacillus licheniformis	Sigma-Aldrich	P5380
Collagenase type 1	Calbiochem	234153
TrypLE™ Express Enzyme (1X), no phenol red	Gibco	12604-013
Paraformaldehye (PFA) 4% in PBS	Thermo Scientific	AAJ19943K2
Dimethyl sulfoxide (DMSO)	Sigma-Aldrich	D8418
dithiobis(succinimidyl propionate) (DSP)	Thermo Scientific	22585
Optiprep Density Gradient Medium	Sigma-Aldrich	D1556
Y-27632	Sigma-Aldrich	Y0503
Mineral Oil	Sigma-Aldrich	M5310
Barcoded Gel Beads - inDrops V2 capture sequence: CGATGACGTAATACGACTCACTATAGGGATACCA CCATGGCTCTTTCCCTACACGACGCTCTTCCGATCT [Barcode 1] GAGTGATTGCTTGTGACGCCTT [Barcode 2] [6bp UMI] T19V	1CellBio	10070
Oil Red O	Alfa Aesar	A12989

**Deposited data**		

Optimization datasets	This study	GEO: GSE234620
K562	Klein et al.^9^	GEO: GSE65525
Fresh and Frozen Human Tumor sc-, sn-RNA-Seq Datasets	Slyper et al.^14^	GEO: GSE140819

**Critical commercial assays**		

MACS® Tumor Dissociation Kit, Mouse	Miltenyi Biotech	130-096-730
MACS® Dead Cell Removal Kit	Miltenyi Biotech	130-090-101

**Experimental models: Organisms/strains**		
C57BL/6J	The Jackson Laboratory	664

**Software and algorithms**		

CellBender	Fleming et al.^22^	https://github.com/broadinstitute/CellBender.git
dropkick	Heiser et al.^18^	https://github.com/Ken-Lau-Lab/dropkick
QCPipe	Chen et al.^42^	https://github.com/Ken-Lau-Lab/STAR_Protocol.git
Scanpy	Wolf et al.^43^	https://github.com/theislab/scanpy
Anndata	Virshup et al.^44^	https://github.com/scverse/anndata.git
Seaborn	Seaborn	https://github.com/mwaskom/seaborn
Matplotlib	Matplotlib	https://github.com/matplotlib/matplotlib
Scipy	Virtanen et al.^45^	https://scipy.org/
Numpy	Harris et al.^46^	https://numpy.org/
Pandas	Pandas	https://pandas.pydata.org/
Scikit-learn	Pedregosa et al.^47^	https://github.com/scikit-learn/scikit-learn.git
AmbiQuant	This study	https://github.com/Ken-Lau-Lab/AmbiQuant

**Other**		

UV Crosslinking oven	Stratagene	Stratalinker 1800
AccuSpin Micro 17R	FisherBrand	13-100-675
Syringe pump	New Era Pump Systems	NE-300
Masterflex tubing	Cole Parmer	MFLX0640660
luer lock connectors	Qosina	80410
Male luer adapter	Idex	P-836
Female luer adapter	Idex	P-835
Primer	Loctite	SF 770
Glue	Loctite	4310
p200 Non-Filter Pipette tips	Biotix	M-0200-9TS
Cell Barcoding Chip	Droplet Genomics	DG-CBC2-80
Droplet Stabilization Oil	Droplet Genomics	DG-DSO-15
100ft Micro medical Tubing .015”I.D. x 0.043”O.D.	Scientific Commodities	BB31695-PE/2
100ft Micro medical Tubing .030”I.D. x 0.048”O.D.	Scientific Commodities	BB31695-PE/4
70μm PluriStrainer Mini Cell Strainers	PluriSelect	43-10070-40
2mL Round bottom tubes	FisherBrand	14-666-315
27G x 0.5" Hypodermic Needles	BD	305109
20G x 1" Hypodermic Needles	BD	305175
Gyromax Orbital Incubator Shaker	Amerex	703
Nexcelom Cellometer Auto 2000	Nexcelom Bioscience	8001334, Rev E
ViaStain™ AO/PI Staining Solutions	Nexcelom Bioscience	CS2-0106-5mL
Cellometer SD100 Counting Chambers	Nexcelom Bioscience	CHT4-SD100-014

### Resource availability

#### Lead contact

Further information and requests for resources and reagents should be directed to and will be fulfilled by the lead contact, Ken Lau (ken.s.lau@vanderbilt.edu).

#### Materials availability

This study did not generate new unique reagents.

### Experimental model and study participant details

Animal experiments were performed under protocols approved by the Vanderbilt University Animal Care and Use Committee and in accordance with NIH guidelines. All animals were housed 2 to 5 per cage in a controlled environment in standard bedding with a standard 12-hour daylight cycle, cessation of light at 7 PM, and free access to standard chow diet and water. Experiments were conducted during the light cycle. Wild-type mice (C57BL/6) of both sexes were euthanized in an approved fashion prior to dissection and tissue harvesting. Mice were generally 6-8 weeks ago at euthanasia.

### Method details

#### Droplet simulation

We used CellBender^22^ to simulate representative datasets of different quality. We generated synthetic datasets by randomizing the number of real cells, number of droplets, number of transcripts from cells, over distributions centered on 2000, 12000, and 5000 respectively (Figures S1A–S1C). In the simulation, a droplet can either contain a cell with simulated biological UMIs or not, where the simulated biological UMIs are generated based on expression profiles pulled from public 10x genomic datasets; the simulated cells have cell-type clusters so that cells share similar biological UMI profile within a cluster and distinct profiles between clusters. All droplets contain some simulated ambient UMIs, defined as a weighted average of total expression, as background. The difference in data quality is specified by the ambient UMI counts in each dataset, which were set to between 5 to 4900 to simulate a range of ambient contamination levels. The number of ambient UMI counts follows a log norm distribution as shown in Figure S1D. We chose the range of 5-4900 ambient UMI because the number of biological UMIs are centered around 5000. A level of 5 ambient UMI per cell confers a ∼0.001 noise-to-signal ratio, which can be considered as an extremely ideal quality in depicting reality. In contrast, a level of 4900 ambient UMI per cell will lead to a 0.98 noise-to-signal ratio, which can be a severe QC failure, beyond which the dataset should strictly be prohibited from further analysis. n=1000 simulations were performed for each ambient level. The parameters of the representative low- and high- contamination datasets in Figures 1A–1D and S1E–S1J are drawn from the same number of real cells, number of droplets, and number of transcripts distributions above, with ambient UMI centered around 100 and 4000 respectively.

#### Sequencing depth simulation

One dataset was simulated for each ambient of the ten ambient levels ranging from 500 to 4900, where ambient levels were the averaged ambient UMI per cell. The simulation was performed using CellBender^22^ as described in the Droplet Simulation section. For each simulated dataset, relative sequencing depth are simulated by randomly downsampling UMI counts from the count matrix to fractions (0.2, 0.4, 0.6, 0.8) of the original dataset’s total UMI counts using scanpy.pp.downsample_counts() function with replacement.

#### Mechanical mincing of tissue

The HTAPP protocol was followed (https://doi.org/10.17504/protocols.io.busfnwbn). Briefly, tissues were minced into 1-2mm pieces using a scalpel followed by enzymatic dissociation outlined below.

#### Crypt isolation by chelation

Colonic crypt isolation was performed.^48^ Briefly, isolated colonic tissues were chelated in buffer consisting of 3 mM EDTA (Corning) and 0.5mM DTT (Teknova) in 1X Dulbecco’s Buffered Saline (DPBS) for 1 hour and 15 minutes rotating at 4°C. The tissue was then transferred to 1X DPBS and shaken rigorously for 2 minutes to separate the colonic epithelium from the tissue. After transfer of crypts to a new tube, shaking was repeated 3X to collect remaining crypts. Crypts were washed in DPBS and divided among various enzymic cocktails and conditions for dissociation.

#### Enzymatic single-cell dissociations

Minced tissues or isolated crypts prepared above were dissociated with various enzymatic protocols (cold protease, MACS enzyme, or DNase1/collagenase cocktail). For dissociation with the cold protease cocktail, tissues were incubated with cold protease (Sigma-Aldrich) (5mg/ml) and Dnase (Sigma-Aldrich) (2.5mg/ml) on a rotator (∼8 rpm) for 25 minutes at 4°C. For enzymatic digestion using the cocktail found in MACs Mouse Tumor dissociation kit (Miltenyi Biotech), tissues were incubated 20 minutes with gentle orbital shaking (∼200RPM) at 37°C. For dissociation using Collagenase (2mg/ml) with Dnase (2.5mg/ml), tissues were incubated for 20 minutes at 37°C static with trituration at 10-minute intervals.^34^ After enzymatic incubations, gentle pipetting with a wide bore p1000 pipette was used to mechanically dissociate tissues, resulting in visibly turbid cell suspensions. After dissociation, the digestion enzyme mixtures were quenched with 2% FBS, and the suspensions were passed through a 70μm filter (Pluriselect) to generate single cells. A series of washes were performed to obtain an optimal single-cell suspension to minimize debris.

For stomach corpus, the mucosa was scrapped off using cell scrapers and incubated in pre-warmed digestion buffer (1mg/mL Collagenase, 2.5mg/ml DNAse) on a 37°C shaker at 220 rpm for 30 minutes. After quenching and filtering, the glands were pelleted at 300 g for 5 minutes and dissociated further in TrypLE (Gibco) and Y-27632 at 37°C for 5 min, and was quenched and spun down at 500g for an additional 5 minutes thereafter prior to encapsulation.

#### Fixation

Single-cell suspensions were fixed with 1% paraformaldehyde (PFA) (Thermo Scientific) or 0.1X DSP (Thermo Scientific). DSP was solubilized in 100% DMSO (Sigma-Aldrich) at a final concentration of 25 mg/mL to form a “25×” stock. 1X DSP solution was prepared by diluting the 25X stock dropwise in 1X DPBS with 2 mM Mg ^2+^ while being continually vortexed and then filtered through a 0.2-μm polyethersulfone membrane syringe filter.^27^ This solution was then added dropwise to single-cell suspensions to achieve the final concentration. Samples were incubated on a rotating platform for 30 minutes at room temperature. Residual DSP was then quenched with Tris-HCl added to a final concentration of 20 mM. Reverse cross-linking was conducted by reducing the disulfide bonds of the DSP fixative using the 10mM DTT present in the standard inDrops RT/Lysis buffer.

#### inDrops encapsulation

The standard inDrops protocol served as the reference scRNA-seq protocol.^49^ inDrops scRNA-seq utilizes CEL-Seq in preparation for sequencing and is summarized as follows: (1) reverse transcription (RT), (2) ExoI nuclease digestion, (3) SPRI purification (SPRIP), (4) single strand synthesis, (5) SPRIP, (6) T7 *in vitro* transcription linear amplification, (7) SPRIP, (8) RNA fragmentation, (9) SPRIP, (10) primer ligation, (11) RT, and (12) library enrichment PCR. We used the TruDrop library structure for sequencing on NovaSeq 6000.^25^ Alignment of reads and barcode deconvolution to generate count matrices was performed using the DropEst pipeline.^50^ The inDrops platform was customized for gut tissues,^32^^,^^51^ and further modifications were made as documented below. Standard loading was performed with assemblies made using 0.38 mm inner diameter tubing 20 cm in length (Scientific Commodities) and fed with a syringe pump. Wide bore loading was performed similarly but with 0.76 mm inner diameter tubing (Scientific Commodities). For enhanced loading, luer lock connectors (Qosina) were spray coated with primer (Loctite) and allowed to dry for 10 minutes in a fume hood. After drying, connectors were glued to pipette tips (Biotix) using UV curing glue (Loctite) and cured using a UV Crosslinker (Stratalinker). Cells were then loaded into the tip assembly, locked using male to female luer adaptors (Idex) to a syringe assembly made using a 30 cm length of tubing (Cole-Parmer), primed with mineral oil (Sigma-Aldrich) colored with oil red (Alfa Aesar), and connected to a syringe pump. Red mineral oil then acted as a void volume to push cells directly into the inDrops encapsulation chip.

#### Dilution microfluidic encapsulation chip

Encapsulations that incorporate dilutions were performed using the “All Cell” chip design as shown in Figure 4I. The chip design was nearly identical to the standard inDrops chip but featured an additional inlet connection to the cell channel for diluting cells immediately prior to entering the encapsulation junction. A syringe pump (New Era Pump Systems) was used to push DPBS into the chip prior to droplet partitioning through this additional channel. Flow rates for cell and dilution buffers were adjusted to total a rate matching that of the reverse transcriptase, such that enzyme and buffer conditions in the final droplets were kept constant.

#### Cell viability enrichment assay

Cells were enriched for viability using the MACS dead cell removal kit (Miltenyi). Cells were incubated with para-magnetic microbeads to label dead and dying cells, then passed through a magnetic column to retain the labeled cells, while yielding a flowthrough of viability enriched cells.

#### Pre-encapsulation tubing comparison

Mouse colon tissue underwent the crypts isolation chelation protocol followed by cold protease enzymatic dissociation protocol. Single cells were then loaded into syringes and passed through either the standard 0.38mm 20 cm tubing or directly from the syringe into Eppendorf tubes in pairwise fashion. Run times remained consistent for each pair. Cells were then mixed in a 1:1 ratio with AO/PI Viability solution then loaded into the Cellometer disposable counting chamber slide and placed into the Cellometer for subsequent counting.

#### Data analysis

##### Data processing

To apply the ambient contamination metrics on a dataset, the first step was to read-in raw gene count data and scale the dataset barcode number relative to the expected real cell number to enable comparison between samples with different numbers of encapsulated cells and empty droplets. The starting point was an unfiltered count matrix that can be of various formats (h5ad, mtx, etc.). An inflection curve was computed using the find_inflection() function from QCPipe.qc module^42^ where a cumulative sum curve of total transcript counts vs barcodes ranked by their transcript counts would be drawn, and the first inflection point of the curve would be used as an estimated real cell number for the sample. This estimation was based on the rationale that, when ambient RNA contamination is not comparable to the true biological transcript counts, droplets capturing real cells contribute to distinctly larger increments in the cumulative count value. In contrast, empty droplets contribute less, so the first inflection point could be a position to approximate captured real cells versus empty droplets. Alternatively, an estimated real cell number could be manually entered in our function as an argument if an expected real cell number for the sample was known. After determining the estimated real cell number, barcodes would be sorted based on their total transcript counts; a threshold would be set as a multiple of the estimated cell number to retain only the high transcript count barcodes beyond the threshold. Through observations of samples used in this study, the multiple was set to 4 as default. This data-processing step could be performed using the cut_off_h5ad() or cut_off_from_dropset() functions from our data_processing python module.

##### Geometric quantification of ambient contamination from the cumulative transcript count curve

Our ambient contamination metric calculation integrated geometric quantifications of the cumulative transcription count curve (Figure 1A). As the total barcode number was set to a multiple of the estimated real cell number, the curve of a high-quality dataset was expected to raise with steep slope in the first portion ($\frac{1}{chosen\;multiple}$), then turn to a relatively flat slope. This shape could cause the curve to deviate notably away from the diagonal linking the final cumulative count and the origin initially (Figure 1A) then gradually get close to the diagonal. However, in low quality cases where ambient RNA molecules keep contributing to high increments of the cumulative count throughout the dataset, the cumulative count curve’s slope would have a low variance, and the curve would not deviate far from the diagonal substantially. Therefore, the magnitude of the curve’s deviation from the diagonal line and the variance within the distances between the curve and the diagonal line are indicators of data quality. We computed the vertical distances between the cumulative sum curve and the diagonal for each barcode, which we defined as secant lines whose maximal value and standard deviation were then calculated to inform data quality. To establish quantitative indicators positively correlated with ambient levels, we inverted the maximal secant distance and the standard deviation by the subtractions:

Invertedmax.secantdistance=1−Max.secantdistance

Invertedsecantlinest.dev.=0.5−secantlinest.dev

The rationale was that the cumulative transcript count curve was normalized to a range of 0 to 1, so all secant lines' length should fall between this range. In extreme cases of secant lines with a maximal distance close to one, a minimal distance close to 0, and a minimal sample size (eg. =3), the standard deviation did not go beyond 0.5 and will always be positive. We used the inverted standard deviation and maximal value of the secant lines as two metrics.

In addition to the secant line distances, as we have described above, a high-quality dataset has a cumulative count curve resembling a rectangular hyperbola with a sharp incline and then flattening of the curve. In contrast, the lack of deviation from the diagonal line for low quality datasets makes the curve resemble a straight line. We quantified the shape differences by first computing the minimal rectangle area circumscribing the cumulative count curve:

Min.rectanglearea=Num.ofbarcodes×Max.cumulativecount

We then computed the area under the cumulative count curve (AUC) using sklearn.metrics.auc() function. Taking the ratio between the area under the cumulative count curve and the area of the minimal rectangle would give a percentage value.

$$AUC\;percentage=\frac{Area\;under\;cumsum\;curve}{\mathrm{Min}.\;rectangle\;area}$$

We inverted the AUC percentage by quantify the area above the curve within the minimal rectangle:

InvertedAUCpercentage=2×(1−AUCpercentage)

A higher inverted AUC percentage value indicates the closeness of the area to the triangle formed from x,y axes and the diagonal, thus high contamination. Therefore, inverted AUC percentage was used as one of our ambient contamination metrics. Steps to compute metrics before inversion were encapsulated in our plot_quality_score.plot_secant_line() function. Alternatively, numerical results alone could be computed from our calculation.secant_metrics() function.

##### Statistical quantification of ambient contamination from the distribution of the slope of the cumulative transcript count curve

As described earlier, high quality datasets have the pattern of a sharp incline followed by flattening of their cumulative transcript count curves, whereas low quality datasets have curves with small change in slopes. The slope difference can be a continuous value informing the quality in a quantitative way. We therefore inspected the slope distribution (Figures S1I and S1J) by generating a histogram on the slopes at each barcode for a sample (Figures S1G and S1H), using the matplotlib.axes.Axes.hist() function, fixing the parameter of number of bins at 100 for consistency. We expected to see a bimodal distribution with a peak contributed by low-slope barcodes followed by a peak contributed by high-slope barcodes, and a higher density at high slope region for high-quality datasets than low-quality datasets was expected. However, even though the two modes could be observed on histograms of high-quality datasets (Figure S1I), it was hard to compare the density values because most droplets in the dataset were expected to be empty due to the way we cut-off the dataset as described above, rendering a notably heavier mass contributed by low-slope barcodes than high-slope barcodes.

To emphasize the density contribution of the high slope barcodes, we transformed the slope distribution to scale up the weights of high-slope barcodes. We performed the transformation by getting each histogram bin’s mean slope value as the x-value for the transformed plot and each bin’s frequency value multiplied by the bin’s mean slope as the y-values for the transformed plot (Figure 1B). In this way, the contribution of distribution density from high-slope barcodes were scaled up based on their slope value, and we were able to quantify the scaled density contributed by these high-slope barcodes as one metric.

To determine slopes that are likely contributed by real cells rather than empty droplets, we set a threshold to have a binary assignment of a barcode to be either real cell or empty droplet. The threshold was calculated as:

Thresholdofslopes=median(slopes)+std(slopes)

Barcodes with slopes higher than the threshold will be identified as real cells, whereas slopes lower than the threshold will be identified as empty barcodes. The rationale behind the calculation of this threshold is due to the slope distribution curve having approximately a bimodal Gaussian distribution (Figure 1B). The majority of slopes (contributed by empty barcodes) lie within the first mode whereas a minority of slopes (contributed by cells) lie within the second mode. The distribution of slopes will always adopt a major and minor peak due to our scaling scheme to always include a multiple of barcodes (4X) of estimated cells in the analysis. Thus, we estimate the center of the low-slope peak as the median of all slopes because the majority of the slopes are captured by the first peak (empty barcodes), with lower percentage of data outside the peak (cells), so we can treat the entire distribution as a single gaussian of the first peak but with outliers that skew the distribution. In this scenario, the median value is a good estimator to reduce the bias caused by the outliers. Using the median + 1 standard deviation captures ∼84% of the data on the left of the cut-off theoretically for a gaussian curve, which is a sufficient threshold to exclude the majority of low-slope datapoints but still leniently keep the datapoints with high slopes. The calculation of this threshold is approximate, but it is notably computationally efficient, and we also verify its robustness with simulation.

We transformed the scaled slope distribution so that the area under the curve summed up to one. Summing the y-values contributed by barcodes beyond or below the threshold gave us the scaled slope contribution from potential real cells and empty droplet respectively. We used the scaled slope sum from the low-slope region (potential empty droplet) as another metric whose value would increase with increased ambient level. Histograms were generated by our plot_quality_score.plot_slope() function, whose return value could be passed to our plot_quality_score. plot_freq_weighted_slope() to generate the scaled slope distribution plot. Alternatively, numerical results alone could be computed from the calculation.freq_slope_area_ratio() function.

##### Ambient gene quantification

Ambient genes were defined as genes that have a dropout rate of less than 2% in this study. We defined ambient RNAs as those transcripts that contaminate all droplets; that is, they should be present in 100% of cells and empty barcodes (dropout rate = 0%). However, given the sensitivity limitation of scRNA-seq, the probability of detecting a gene when it is present in a droplet follows binomial sampling statistics and is proportional to its expression level. A high expressing gene (those that have > 45 transcripts per droplet) can be detected ∼95% (5% dropout rate) of time.^9^ Since ambient transcript count varies depending on the level of contamination, we set a slightly stricter threshold (2% dropout rate) so that only highly prevalent transcripts are defined as ambient. The dropout rate is also a parameter in the pipeline that end-users can vary. Percentage counts of ambient genes were computed with scanpy.pp.calulate_qc_metrics() function, with the specific argument ‘qc_vars = [“ambient”]’, where ‘ambient’ was an anndata object’s obs variable composed of a list of boolean labels identifying the ambient genes among all genes. Histograms of the distribution of percentage counts of ambient genes were made with seaborn.histplot() function.

##### Overall score calculation

To assess the contribution of each ambient contamination metrics in depicting the ambient contamination degree of a given dataset, we performed a principal component analysis (PCA) on metric scores of simulated data (Droplet Simulation Section) with n = 10,000 datasets and 6 features (empty droplet slope sum, inverted max. secant line distance, inverted secant line stdev, inverted AUC percentage, number of ambient genes, averaged percent counts ambient).

Scikit-learn package was used to perform the PCA.^47^ We transformed the score matrix using the fit_transform() function called from a StandardScaler object from the sklearn.prepocessing module. A PCA object from the sklearn.decomposition module was initiated with the parameter n_components=6. PCA was computed using the fit_transform() function called from the PCA object with the transformed score matrix as the input.

Based on the result of the PCA, we calculated the overall score as a weighted linear combination of the empty droplet slope sum, inverted max. secant line distance, transformed inverted secant line stdev, inverted AUC percentage, and averaged percent counts ambient. The inverted secant line st. dev. values are transformed by doubling the original values so that all metric scores used in calculating the overall score are bounded between 0 and 1.

##### Standard quality control metrics calculation

We followed the steps of^42^ to filter barcodes based on dropkick scores, standard QC information and biological markers. Total transcript counts, total counts of gene detected and percentage counts of mitochondrial genes were calculated with scanpy.pp.calulate_qc_metrics() function on datasets after filtering and were used as the standard QC metrics in our study.

##### UMAP visualization

For single-cell RNA-seq data, we normalized raw count data by median library size, log-like transformed with Arcsinh, and Z- score standardized per gene using scanpy and numpy functions. For the metrics scores matrix, we performed the Arcsinh transformation and Z-score standardization with scanpy and numpy functions. UMAP coordinates were calculated after PCA and KNN clustering on the matrices as described in^42^ and UMAPs were visualized with scanpy.pl.umap() function.

##### Heatmap and 3-D scatterplot visualization

The heatmap was generated using seaborn.clustermap() function with the input of matrix of the 6 ambient contamination metric and the 4 standard QC metric scores as rows and the HTAPP samples as columns. The matrix was sorted by samples' Leiden cluster labels and then by sequencing technique & protocol labels before making the heatmap; 'col_cluster' and 'row_cluster' were set to false as the function's input. Metadata of sequencing techniques, sequencing technniques & protocols, sample types, cell origins and cancer types were input as a list of mapped colors as the col_colors argument to the function. The 3-D scatter plot was generated with matplotlib.Axes.scatter() function; input x,y,z coordinates were the 3 principal components of the HTAPP sample's ambient contamination and standard QC metric scores after Principal Component Analysis (PCA). PCA was performed using scanpy.tl.pca() function with svd_solver = 'arpack' argument after the metric score matrix was archsinh transformed and scaled to unit variance and 0 mean using numpy and scanpy functions.

### Quantification and statistical analysis

p-values from two-sided, unpaired t-test and One-way ANOVA, Tukey post-test. p-values below 0.05 are considered statistically significant.

## Data Availability

• Single-cell RNA-seq data have been deposited at NCBI GEO and are publicly available as of the date of publication with accession number GEO: GSE234620. • Code generated in this work for the contamination metrics pipeline has been deposited at Github and is publicly available as of the date of publication: https://github.com/Ken-Lau-Lab/AmbiQuant.git • Any additional information required to reanalyze the data reported in this paper is available from the lead contact upon request.
