# Supplementary material for: A contamination focused approach for optimizing the single-cell RNA-seq experiment
Source: iScience. 2023 Jun 29;26(7):107242. doi: 10.1016/j.isci.2023.107242 (PMC10366499; doi:10.1016/j.isci.2023.107242)
Supplement: Document S1. Figures S1–S5 and Tables S1 and S2 [file mmc1.pdf]

iScience, Volume *26*

Supplemental information

A contamination focused approach for optimizing the single-cell RNA-seq experiment

Deronisha Arceneaux, Zhengyi Chen, Alan J. Simmons, Cody N. Heiser, Austin N. Southard-Smith, Michael J. Brenan, Yilin Yang, Bob Chen, Yanwen Xu, Eunyoung Choi, Joshua D. Campbell, Qi Liu, and Ken S. Lau

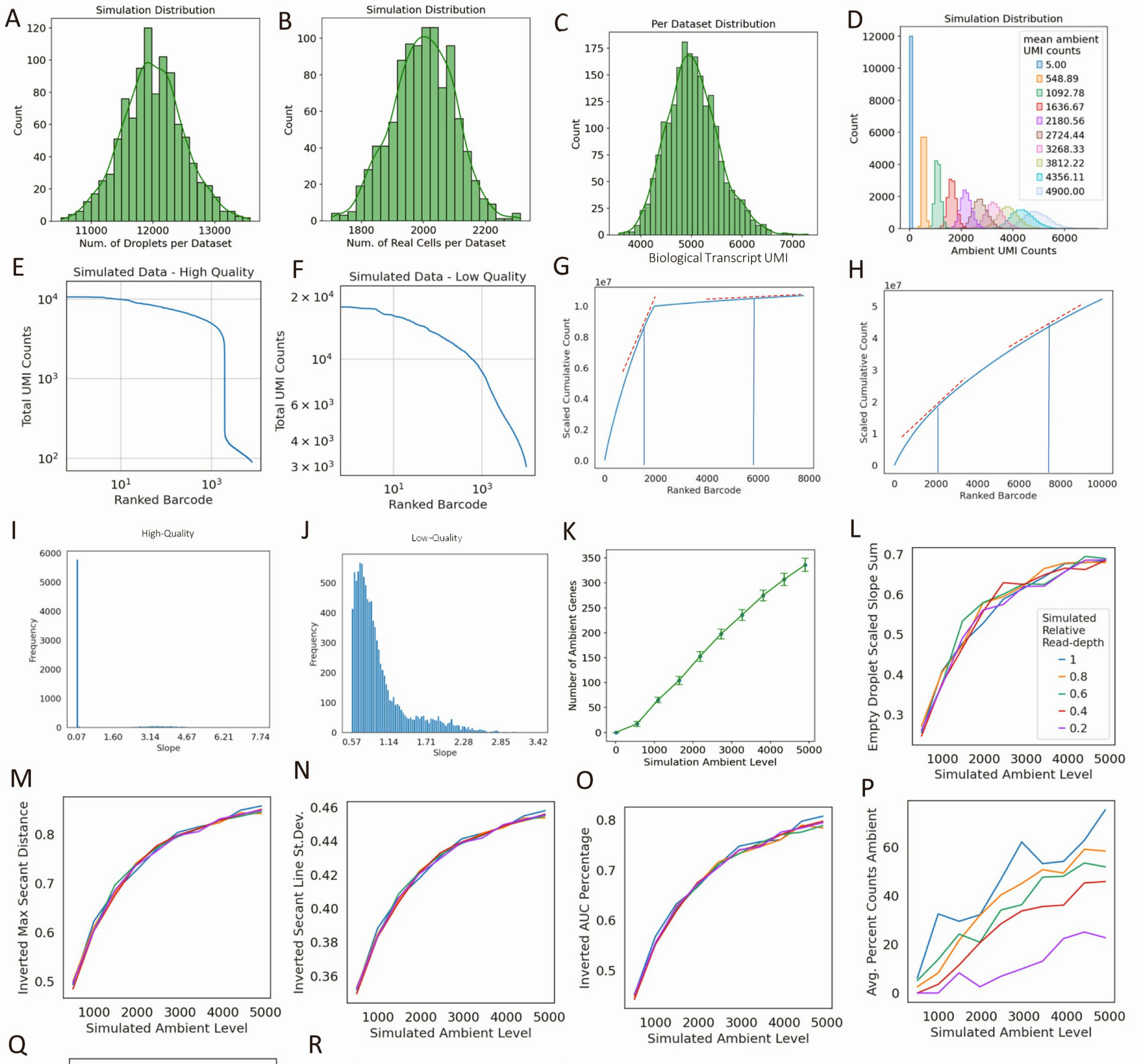

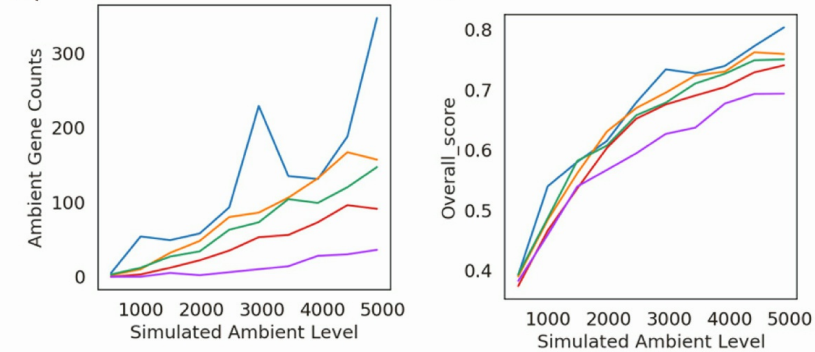

Fig. S1. Ambient contamination metrics of data simulated with different sequencing depth and ambient contamination levels. Related to Fig. 1. **(A-B)** Distribution of the number of **(A)** droplets and **(B)** real cells per dataset for 1000 datasets simulated by CellBender. **(C)** Distribution of the number of biological transcripts UMI counts per cells for a dataset with ~2000 cells simulated by CellBender. **(D)** Distribution of the number of ambient UMI counts per droplets for various datasets at different ambient levels. 120,000 droplets per dataset simulated by CellBender. **(E-F)** UMI count versus log ranked barcodes curve for datasets simulated with **(E)** low ambient level and **(F)** high ambient level. **(G-H)** Scaled cumulative total transcript counts over ranked barcodes by total transcript counts for datasets simulated with **(G)** low ambient level and **(H)** high ambient level. Slopes (dashed pink lines) distinguishing real cells and empty droplets can be observed in **(G)**, but not in **(H)**. **(I-J)** Distribution of the slopes of the four curves shown in **(E-F)**, respectively. **(K)** Number of ambient genes over ambient levels as defined in STAR Methods for simulations. X- axes are ambient levels, which are the averaged ambient UMI counts per cell for a simulated sample. Line plots shown as mean ± stdev of n=1000 replicates for each ambient level. **(L-R)** Simulated datasets with different sequencing depths (line colors) and contamination levels (X-axis) to demonstrate effects on contamination metrics: **(L)** Empty Droplet Scaled Slope Sum, **(M)** Inverted Max. Secant Distance, **(N)** Inverted Secant Line Standard Deviation, **(O)** Inverted AUC Percentage, **(P)** Averaged Percent Counts Ambient, **(Q)** Ambient Gene Counts, and **(R)** Overall Score.

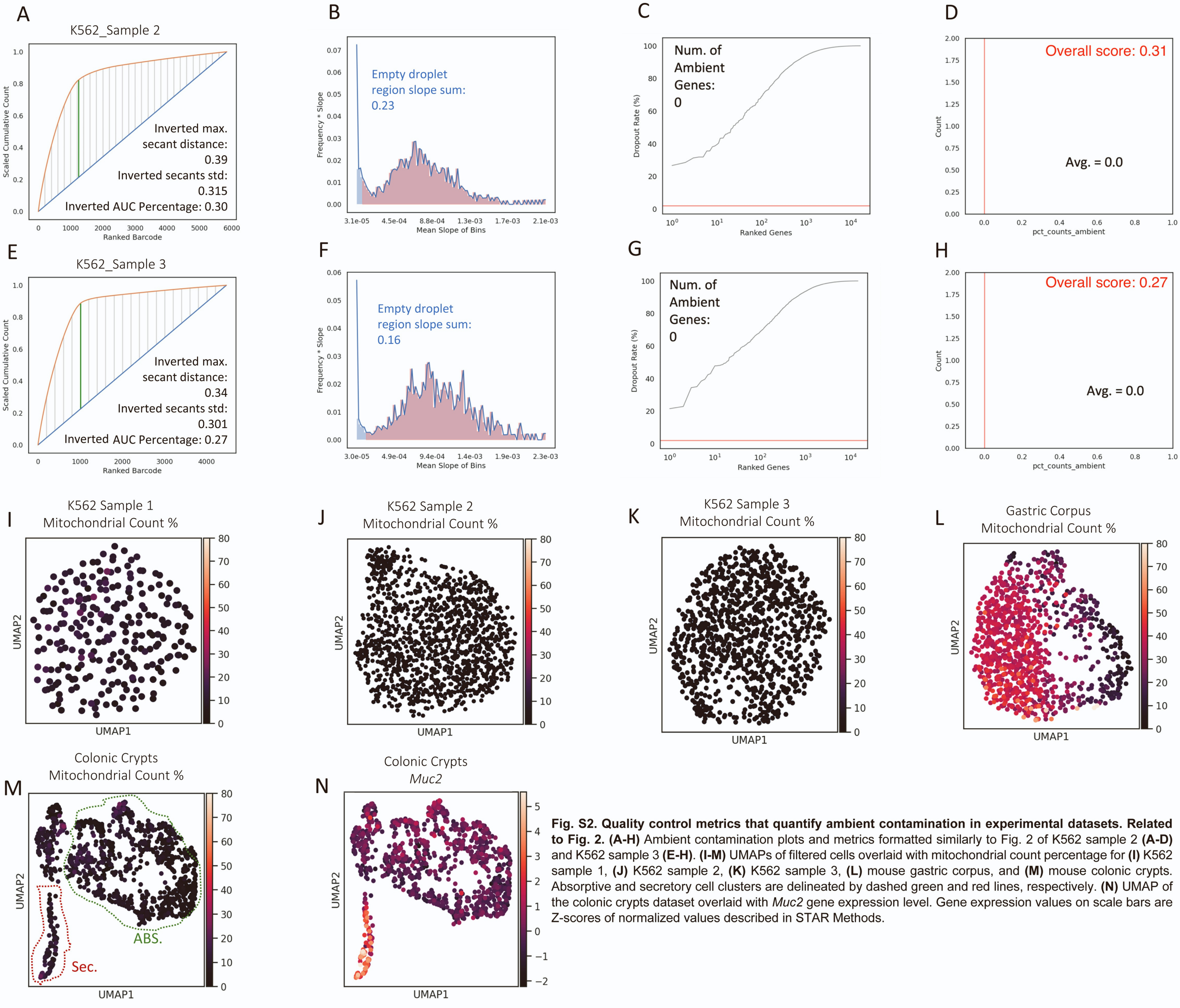

Fig. S2. Quality control metrics that quantify ambient contamination in experimental datasets. Related to Fig. 2. (A-H) Ambient contamination plots and metrics formatted similarly to Fig. 2 of K562 sample 2 **(A-D)** and K562 sample 3 **(E-H)**. **(I-M)** UMAPs of filtered cells overlaid with mitochondrial count percentage for **(I)** K562 sample 1, **(J)** K562 sample 2, **(K)** K562 sample 3, **(L)** mouse gastric corpus, and **(M)** mouse colonic crypts. Absorptive and secretory cell clusters are delineated by dashed green and red lines, respectively. **(N)** UMAP of the colonic crypts dataset overlaid with *Muc2* gene expression level. Gene expression values on scale bars are Z-scores of normalized values described in STAR Methods.

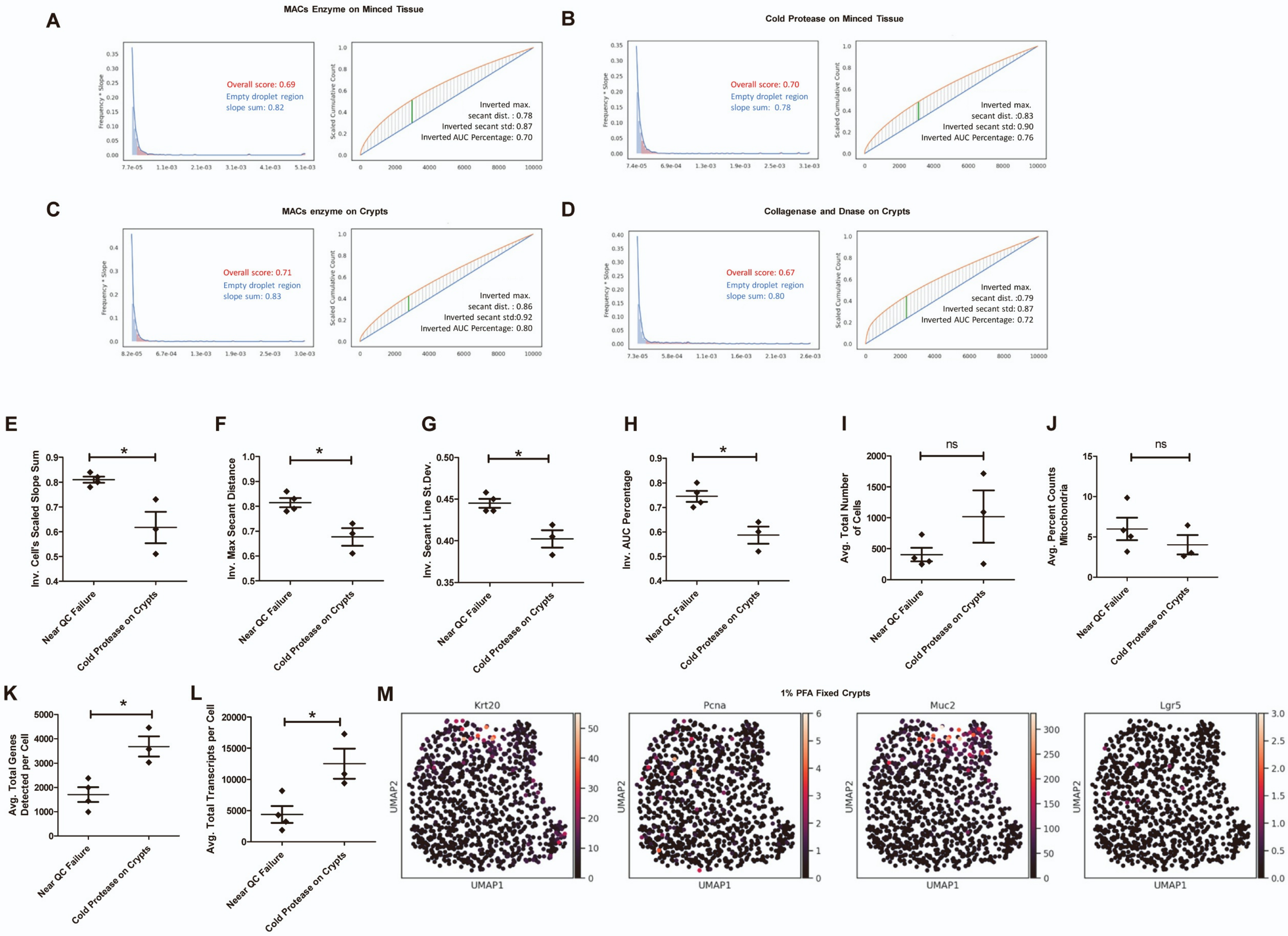

Fig. S3. Quantitative assessment pre-encapsulation factors that affect data quality. Related to Fig. 3. (A-D) Ambient contamination plots formatted similarly to Fig. 1 derived from colonic datasets generated using various dissociation protocols, showing near QC failure (MACs enzyme on minced tissue, cold protease on minced tissue, MACs enzyme on crypts, and Collagenase/Dnase on crypts). **(E-L)** Quantification of **(E-H)** contamination and **(I-L)** standard metrics comparing near QC failure runs and cold protease dissociation on crypts. Mean with SEM as error bars for n=3 or 4 samples. *p<0.05 by t-test. **(M)** UMAP visualization colored according to gene expression for cell type markers for 1% PFA fixation colonic crypt scRNA-seq dataset. Gene expression values on scale bars are Z-scores of normalized values described in STAR Methods.

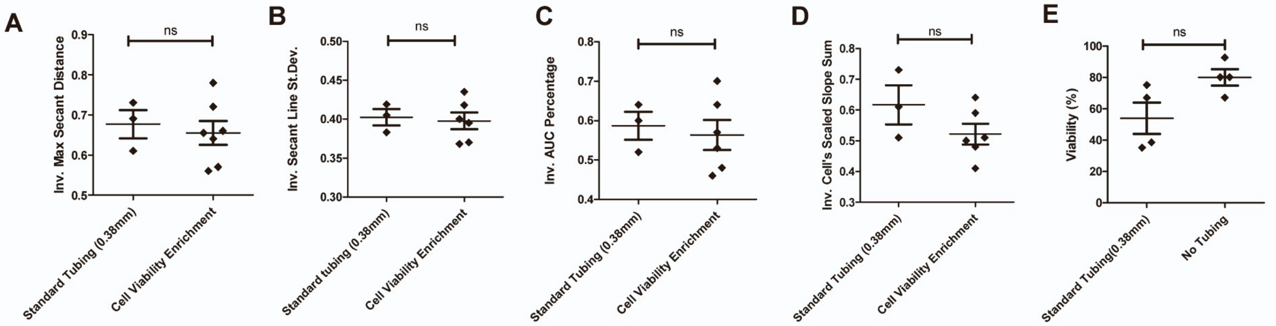

F

Avg. Total Number of Cells					
Tukey's Multiple Comparison Test	Mean Diff.	q	Significant? P < 0.05?	Summary	95% CI of diff
Tip Loading (0.51mm) vs Standard tubing (0.38mm)	1861	5.323	Yes	*	448.3 to 3274
Tip Loading (0.51mm) vs All Cell	2503	7.732	Yes	**	1195 to 3811
Standard tubing (0.38mm) vs All Cell	641.7	1.835	No	ns	-771.1 to 2054

Avg. Percent Counts Mitochondria					
Tukey's Multiple Comparison Test	Mean Diff.	q	Significant? P < 0.05?	Summary	95% CI of diff
Tip Loading (0.51mm) vs Standard tubing (0.38mm)	0.2208	0.2624	No	ns	-3.180 to 3.622
Tip Loading (0.51mm) vs All Cell	0.225	0.2887	No	ns	-2.924 to 3.374
Standard tubing (0.38mm) vs All Cell	0.004167	0.00495	No	ns	-3.397 to 3.405

Average Total Genes Detected					
Tukey's Multiple Comparison Test	Mean Diff.	q	Significant? P < 0.05?	Summary	95% CI of diff
Tip Loading (0.51mm) vs Standard tubing (0.38mm)	-878.6	3.871	No	ns	-1796 to 38.60
Tip Loading (0.51mm) vs All Cell	404.3	1.924	No	ns	-444.9 to 1254
Standard tubing (0.38mm) vs All Cell	1283	5.652	Yes	**	365.7 to 2200

Average Total Transcripts					
Tukey's Multiple Comparison Test	Mean Diff.	q	Significant? P < 0.05?	Summary	95% CI of diff
Tip Loading (0.51mm) vs Standard tubing (0.38mm)	-4807	3.431	No	ns	-10468 to 854.3
Tip Loading (0.51mm) vs All Cell	141.8	0.1093	No	ns	-5099 to 5383
Standard tubing (0.38mm) vs All Cell	4949	3.532	No	ns	-712.5 to 10610

Percent Counts Ambient					
Tukey's Multiple Comparison Test	Mean Diff.	q	Significant? P < 0.05?	Summary	95% CI of diff
Tip Loading (0.51mm) vs Standard tubing (0.38mm)	-12.46	5.202	Yes	*	-22.14 to -2.781
Tip Loading (0.51mm) vs All Cell	-0.375	0.1691	No	ns	-9.334 to 8.584
Standard tubing (0.38mm) vs All Cell	12.08	5.046	Yes	*	2.406 to 21.76

Inverted Cell's Scaled Slope Sum					
Tukey's Multiple Comparison Test	Mean Diff.	q	Significant? P < 0.05?	Summary	95% CI of diff
Tip Loading (0.51mm) vs Standard tubing (0.38mm)	-0.3067	7.634	Yes	**	-0.4690 to -0.1443
Tip Loading (0.51mm) vs All Cell	-0.23	6.184	Yes	**	-0.3803 to -0.07970
Standard tubing (0.38mm) vs All Cell	0.07667	1.908	No	ns	-0.08567 to 0.2390

Inverted Max Secant Distance					
Tukey's Multiple Comparison Test	Mean Diff.	q	Significant? P < 0.05?	Summary	95% CI of diff
Tip Loading (0.51mm) vs Standard tubing (0.38mm)	-0.2142	6.453	Yes	**	-0.3483 to -0.08004
Tip Loading (0.51mm) vs All Cell	-0.07	2.278	No	ns	-0.1942 to 0.05418
Standard tubing (0.38mm) vs All Cell	0.1442	4.344	Yes	*	0.01004 to 0.2783

Inverted Secant Line St.Dev					
Tukey's Multiple Comparison Test	Mean Diff.	q	Significant? P < 0.05?	Summary	95% CI of diff
Tip Loading (0.51mm) vs Standard tubing (0.38mm)	-0.06508	6.376	Yes	**	-0.1063 to -0.02383
Tip Loading (0.51mm) vs All Cell	-0.0185	1.958	No	ns	-0.05669 to 0.01969
Standard tubing (0.38mm) vs All Cell	0.04658	4.563	Yes	*	0.005333 to 0.08783

Inverted AUC Percentage					
Tukey's Multiple Comparison Test	Mean Diff.	q	Significant? P < 0.05?	Summary	95% CI of diff
Tip Loading (0.51mm) vs Standard tubing (0.38mm)	-0.2367	5.648	Yes	**	-0.4060 to -0.06733
Tip Loading (0.51mm) vs All Cell	-0.045	1.16	No	ns	-0.2018 to 0.1118
Standard tubing (0.38mm) vs All Cell	0.1917	4.574	Yes	*	0.02233 to 0.3610

Overall Score					
Tukey's Multiple Comparison Test	Mean Diff.	q	Significant? P < 0.05?	Summary	95% CI of diff
Tip Loading (0.51mm) vs Standard Tubing (0.38mm)	-0.2011	7.304	Yes	**	-0.3123 to -0.08983
Tip Loading (0.51mm) vs All Cell	-0.07562	2.967	No	ns	-0.1786 to 0.02738
Standard Tubing (0.38mm) vs All Cell	0.1255	4.557	Yes	*	0.01421 to 0.2367

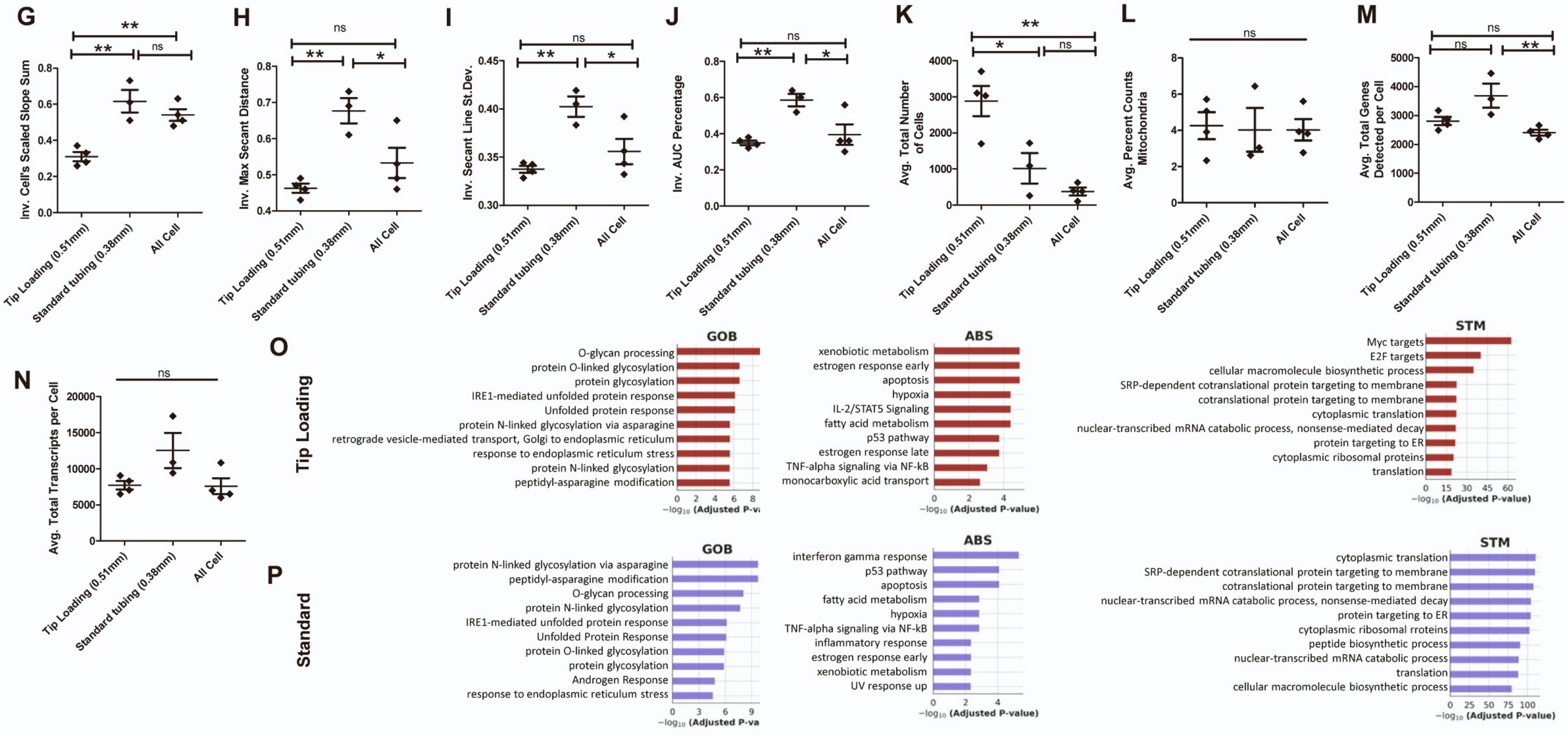

Fig. S4. Evaluation of post-dissociation factors in affecting data quality. Related to Fig. 4 (A-D) Contamination metrics on standard tubing and standard tubing post cell viability enrichment datasets. Mean with SEM as error bars for n=3 or 6 samples.**(C)**Cell viability of cells exiting 20 cm of 0.38mm tubing or directly from the syringe pump. **(F)** Table of Tukey's post-test values calculated for each metric. **(G-N)** Quantification of **(G-J)** contamination and **(K-N)** standard metrics comparing various microfluidics manipulations. Mean with SEM as error bars for n=3 or 4 samples. *p<0.05, **p<0.01 by ANOVA followed by Tukey post-test. **(O-P)** Comparison of functional enrichment analysis datasets derived from **(O)** tip loading (higher data quality) and **(P)** standard loading (lower data quality) looking at Goblet (GOB), absorptive (ABS) and stem (STM) cells.

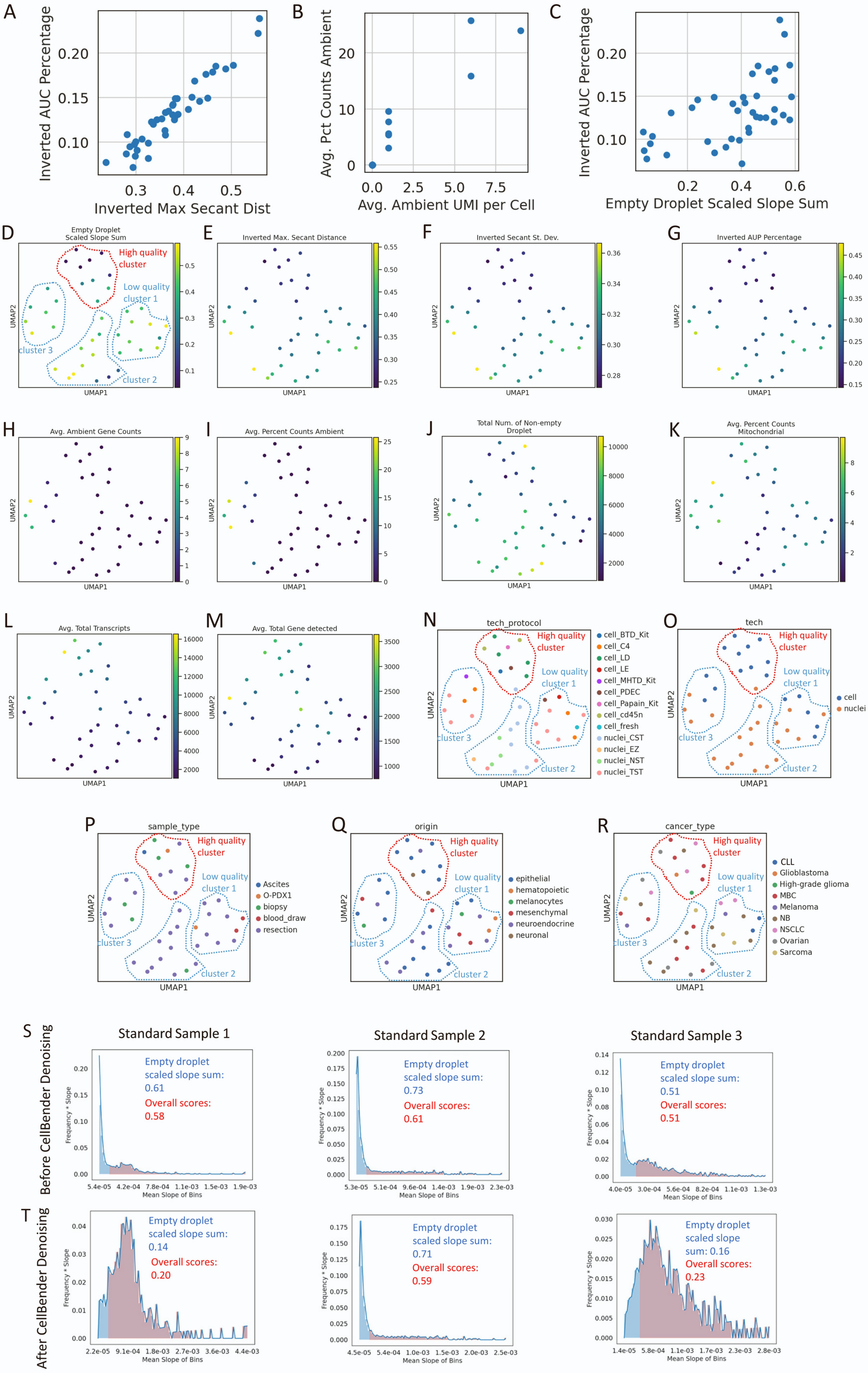

Fig. S5. Ambient contamination, quality control metrics and sample metadata visualization. Related to Fig. 5

(A-C) Correlation of **(A)** Inverted Max Secant Dist vs. Inverted AUC Percentage, **(B)** Avg. Ambient UMI per Cell vs. Avg. Pct Counts Ambient, and **(C)** Empty Droplet Scaled Slope Sum vs. Inverted AUC Percentage. **(D-R)** UMAPs of ambient contamination and standard QC metric scores overlaid with **(D)** empty droplet slope sum, **(E)** inverted maximal secant distance, **(F)** inverted secant line standard deviation, **(G)** inverted AUC percentage, **(H)** average ambient gene counts, **(I)** average percentage counts of ambient genes, **(J)** total number of cells **(K)** average percentage counts of mitochondrial genes, **(L)** average total transcript counts per cell, **(M)** average total gene per cell, **(N)** isolation technique, **(O)** technique x protocol combination, **(P)** sample type, **(Q)** tissue origin, **(R)** cancer type. The high-quality cluster is circled in red dash line, and low-quality clusters are circled in blue dash lines. Abbreviations same as Fig.5. **(S)** Scaled Slope sum contamination metric of 3 low quality datasets before CellBender Denoising. **(T)** Resultant Scaled Slope Sum contamination metrics applied to the same datasets in S after CellBender Denoising.

Table S1. Quality Control Metrics on Experimental Data with Various Degrees of Quality. Related to Table 1.

	K562 Sample 1	K562 Sample 2	K562 Sample 3	Cold Protease on Crypts	Gastric Corpus
Overall Score	0.25	0.31	0.27	0.58	0.78
**Contamination Metrics**					
Empty Droplet Scaled Slope Sum	0.17	0.15	0.11	0.61	0.75
Inverted Max Secant Distance	0.28	0.39	0.34	0.69	0.86
Inverted Secant Line St.Dev.	0.28	0.32	0.30	0.41	0.46
Inverted AUC Percentage	0.20	0.30	0.26	0.60	0.80
Avg. Percent Counts Ambient	5.50	0.00	0.00	18.26	56.89
**Standard Metrics**					
Total Number of Cells	239.00	1248.00	1009.00	1090.00	1000.00
Avg. Percent Counts Mitochondria	9.62	0.65	0.74	6.43	31.60
Avg.Total Genes per Cell	28061.28	1994.62	2362.86	9395.47	1542.61
Avg. Total Transcripts per Cell	7330.51	841.00	897.48	3026.36	453.61

Table S2. Quality Control Metrics on Minced Tissue Dissociation under Different Loading Condition. Related to Table 2.

	Tip Loading on Minced Tissue	MACS Enzyme on Minced Tissue with Standard Tubing	Cold Protease on Minced Tissue with Standard Tubing
**Overall Score**	0.44	0.69	0.70
**Contamination Metrics**			
Empty Droplet Scaled Slope Sum	0.51	0.82	0.78
Inverted Max Secant Distance	0.54	0.78	0.83
Inverted Secant Line St.Dev.	0.36	0.44	0.45
Inverted AUC Percentage	0.42	0.70	0.76
Avg. Percent Counts Ambient	2.86	27.06	22.34
**Standard Metrics**			
Total Number of Cells	1032.00	730.00	295.00
Avg. Percent Counts Mitochondria	4.11	9.86	5.06
Avg.Total Genes per Cell	2068.38	990.00	1434.31
Avg. Total Transcripts per Cell	4809.52	1860.70	3220.45
